# Synthetic β-sheets mimicking fibrillar and oligomeric structures for evaluation of spectral X-ray scattering technique for biomarker quantification

**DOI:** 10.1186/s13578-024-01208-6

**Published:** 2024-02-19

**Authors:** Karthika Suresh, Eshan Dahal, Aldo Badano

**Affiliations:** Division of Imaging, Diagnostics, and Software Reliability, Office of Science and Engineering Laboratories, Center for Devices and Radiological Health, Food and Drug Administration, Silver Spring, MD 20993 USA

**Keywords:** Neurodegenerative diseases, Biomarker, Protein aggregates, β-sheet, Cross-β, Oligomer, Fibril, X-ray scattering

## Abstract

**Background:**

Archetypical cross-β spines sharpen the boundary between functional and pathological proteins including β-amyloid, tau, α-synuclein and transthyretin are linked to many debilitating human neurodegenerative and non-neurodegenerative amyloidoses. An increased focus on development of pathogenic β-sheet specific fluid and imaging structural biomarkers and conformation-specific monoclonal antibodies in targeted therapies has been recently observed. Identification and quantification of pathogenic oligomers remain challenging for existing neuroimaging modalities.

**Results:**

We propose two artificial β-sheets which can mimic the nanoscopic structural characteristics of pathogenic oligomers and fibrils for evaluating the performance of a label free, X-ray based biomarker detection and quantification technique. Highly similar structure with elliptical cross-section and parallel cross-β motif is observed among recombinant α-synuclein fibril, Aβ-42 fibril and artificial β-sheet fibrils. We then use these β-sheet models to assess the performance of spectral small angle X-ray scattering (sSAXS) technique for detecting β-sheet structures. sSAXS showed quantitatively accurate detection of antiparallel, cross-β artificial oligomers from a tissue mimicking environment and significant distinction between different oligomer packing densities such as diffuse and dense packings.

**Conclusion:**

The proposed synthetic β-sheet models mimicked the nanoscopic structural characteristics of β-sheets of fibrillar and oligomeric states of Aβ and α-synuclein based on the ATR-FTIR and SAXS data. The tunability of β-sheet proportions and shapes of structural motifs, and the low-cost of these β-sheet models can become useful test materials for evaluating β-sheet or amyloid specific biomarkers in a wide range of neurological diseases. By using the proposed synthetic β-sheet models, our study indicates that the sSAXS has potential to evaluate different stages of β-sheet-enriched structures including oligomers of pathogenic proteins.

**Supplementary Information:**

The online version contains supplementary material available at 10.1186/s13578-024-01208-6.

## Introduction

Worldwide, neurodegenerative diseases (NDDs) affect millions and the estimated global number of patients with dementia now exceeds 50 million [1, 2]. These NDDs include Alzheimer’s disease (AD), Parkinson’s disease (PD), frontotemporal dementia (FTD), prion diseases (PrDs) and amyotrophic lateral sclerosis (ALS). The onset of neurodegeneration is instigated by key events, including protein misfolding, aggregation in the form of oligomers and fibrils and subsequent accumulation in the brain. These processes collectively contribute to the emergence of pathological abnormalities responsible for neurodegenerative disorders.[3, 4]. The proteins incriminated in the accumulation of cerebral misfolded aggregates include amyloid β (Aβ) in AD; tau in AD and FTD; α- synuclein (α- Syn) in PD; prion proteins (PrPs) in PrDs and TAR DNA-binding protein 43 (TDP-43) in ALS [5–9].

The pathogenic fibrillar aggregates of Aβ, tau, α-Syn, PrP and TDP-43 comprise a structural superfamily and share a common substructure at the nanometer length scale, although they do not share sequence homology or related native structure [10]. The characteristic features of these pathogenic aggregates are summarized in Table 1. Aberrant native proteins follow a hierarchical aggregation pathway where the secondary structure of peptides alter its folds from healthy disordered or α-helical to pathological β-sheet enriched structures ranging from soluble toxic oligomers to protofibrils to large fibrillar plaques or tangles. Oligomers are spherical or annular and increase in oligomer size leads to increase in compactness, β-sheet content, structural regularity, stability and hydrophobic surface burial [11]. End-stage, mature, insoluble aggregates take the structural form of thin, unbranched fibrils only a few nanometers in diameter but often micrometers in length. They exhibit a cross-β structural pattern containing ribbon-like β-sheets in which the β-strand segments run perpendicular to the fiber major axis and intermolecular hydrogen bonds of β-strands run parallel to the axis [12]. Generic cross-β structural motif was also reported for pathogenic fibrils of non-NDD amyloidoses as exemplified by amyloid light-chain (AL) amyloidosis, transthyretin (TTR) amyloidosis [13]. In this case, the aggregate depositions can occur across different organs and biological barriers in vivo including spleen, kidneys, liver and heart.

**Table 1:** Summary of reported secondary structures and features of nanoscale structures of various pathogenic proteins in neurodegenerative diseases

Protein	β-Amyloid plaque	α- synuclein Lewy body	Tau tangle	Prion	TDP-43
Disease referring	AD	PD	Down’s syndrome	CJD, TSE	FTLD-TDP
Major secondary structure	β-sheet	β-sheet	β-sheet	β-sheet [38]	β-sheet
β-sheet IR absorption wavenumber (cm^−1^)	1632 [39], 1628 [40]	1628, 1680 [41]	1628 [42, 43]	1630, 1628, 1618, 1614 [44]	1626 [45]
% β-sheet	53 [40, 46, 47]	LB core: 49 LB halo: 64 [41]	57 [48]	36–42, 54^a^ [49, 50]	52 [51] Wild type: 40 [45]
Conformation, X-ray peak positions (nm)	cross-β, 0.476, 1.06 [52]	cross-β, 0.47, 1 [12, 53]	cross-β, 0.47, 1.3 [48]	cross-β [54], 0.48,1.05^a^ [55]	cross-β^b^, 0.48, 1.0^c^ [56, 57]
Reported filament diameters (nm)	10–20 [58–60]	5–20 [61–63]	8–20 [23]	9–20 [64–67]	15–25 [57, 68, 69]

^a^For scrapie PrP [49], ^b^Some studies have also reported that not all TDP-43 aggregates have cross β-sheet structures [70], ^c^In-vitro fibrillization, FTLD-TDP: frontotemporal lobar degeneration with TPD-43-immunoreactive pathology, CJD: Creutzfeldt-Jakob disease, TSE: transmissible spongiform encephalopathies

The characteristic tinctorial properties and cross-β diffraction pattern are diagnostic hallmarks of various NDDs [14–16]. Historically, the neuropathological lesions are identified with high certainty by histopathological examination at autopsy. Here, the protein aggregates are selectively stained with histological dyes such as thioflavin-S and Congo red which bind the cross-β structure [14]. Conformation-specific antemortem structural biomarkers are also gaining rapid interest for the detection of different stages of β-sheet-enriched structures [17–20]. In AD, Aβ aggregation into β-sheet-rich structures occur up to 15–20 years prior to clinical manifestation and causes amyloid plaque deposition in the brain. Therefore, research diagnostic criteria in AD recommends amyloid-based biomarkers [21, 22]. The change to an increased β-sheet structure of Aβ aggregates is correlated with cerebrospinal fluid (CSF) AD biomarkers and amyloid positron emission tomography (PET) imaging [15, 17]. The change in secondary structure of Aβ in human blood is also reported as a blood-based amyloid indicator for prodromal AD [16]. Many β-sheet binding ligands including radiolabeled small molecule dyes such as thioflavin and stilbene derivatives have been reported through the development of amyloid PET tracers [23]. Additionally, first- and second-generation tau tracers are also based on the detection of specific binding to the β-sheet regions of aggregated tau [24]. The density of binding sites of α-syn and TDP-43 aggregates are lower than Aβ and tau, posing challenge to the development of novel PET tracers for α-syn and TDP-43 [18, 25]. Moreover, chemical and pharmacological properties requirements including blood–brain barrier permeability, high binding affinity and specificity to targets and ability to undergo rapid clearance from normal brain tissue and blood along with high cost and time further add challenges to PET imaging.

Seed amplification assay (SAA) is another reported fluid-based biomarker test of pathology mainly for prion disease and synucleinopathies including PD characterized by protein aggregates [19]. This technique amplifies very small amounts of misfolded aggregates of proteins to the point that can be detected using standard laboratory techniques. A recent CSF-based large cross-sectional study showed that the SAA can classify people with Parkinson’s disease with high sensitivity and specificity [20]. This assay is in its developmental stage, and standardization of seed characteristics and aggregation protocol, along with the evaluation of its prognostic value by a longitudinal study, are yet to be established. Non-visualization of regional pathogen distribution adds to its limitations.

Spectral small angle X-ray scattering (sSAXS) is an emerging label-free biomarker quantification tool to detect various β-sheet rich protein aggregates using X-rays [26–31]. This noninvasive technique, comprised of a polychromatic X-ray beam and a spectroscopic photon-counting detector, is shown to capture elastic X-ray scattering signatures of β-sheet structure [26–29]. Preliminary investigations have demonstrated the viability of the sSAXS-based approach in phantoms and mice studies. In contrast to the conventional SAXS, sSAXS allows the imaging of objects as thick as a human head [31]. Brain amyloid burden in AD mice model estimated by sSAXS correlated well with the gold standard histological results [26].

One of the major challenges with the development and optimization of new biomarker quantification techniques for NDDs including sSAXS is the scarcity of human derived pathogenic proteins and high cost of recombinant proteins [32]. Conversions of non-pathogenic peptides and proteins, into aggregates with the characteristics of pathogenic amyloid fibrils have been reported previously [33–37]. Even though, these models qualitatively mimic Aβ-fibrils but often fail to quantitatively match the β-sheet secondary structure. In this present study, we first aim to tune the secondary structure and nanoscopic structures of two protein models to match the % β-sheet content and structures of X-ray scatterers within the length scale of 0.25–45 nm with the NDD related protein aggregates. Mimicking the structures on the farther side of this length scale is beyond the scope of this study. The definition of synthetic aggregates in this study relies on the β-sheet content and X-ray scattering properties within the wavevector range of *q* = 0.14 nm^−1^ to 25 nm^−1^, corresponding to structural length scales from 0.25 to 45 nm. In this study, the requisite criteria for synthetic fibrils included the ability to form fibrils with elliptical cross-sections, parallel β-sheets, and a cross-β structure with a parallel arrangement of β-sheets with respect to the fibril’s major axis. Similarly, the criteria for synthetic oligomers included the presence of a β-sheet secondary structure with a β-sheet content intermediate to that of functional monomers (% β-sheet: 0) and matured fibrils. Additionally, they were required to have antiparallel β-sheets with a high oligomer index (OI > 0.2), an ellipsoidal shape with dimensions comparable to oligomers of NDDs, and an exhibited cross-β X-ray scattering profile.

Here, we engineered the self-assembly of two distinct hydrolyzed globular proteins-bovine serum albumin and β-lactoglobulin to mimic the geometry, secondary structure and β-sheet content of fibrillar and prefibrillar states of Aβ-42. We then assessed the β-sheet detection and quantification performance of sSAXS using these β-sheet mimicking model proteins. Quantitative evaluation of the sSAXS performance under brain tissue environment which mimics the tissue X-ray attenuation characteristics is also performed using two component phantoms. The tunability of β-sheet proportions and shapes of structural motifs, and the low-cost of these β-sheet models can become useful test materials for evaluating β-sheet or amyloid specific biomarkers in a wide range of neurological diseases.

## Materials and methods

The recombinant α-synuclein pre-formed fibrils (catalog number- ASF-1001-1) were purchased from rPeptide (Watkinsville, GA). The preformed fibrils were dispersed in buffer solution of pH 7.4 with counter ions of 200 mM tris–HCl, 25 mM NaCl and with 1 wt.% concentration. The Aβ-42 fibrils (SQ-ANAK-80928) were purchased from AnaSpec (Fremont, CA). β-lactoglobulin from bovine milk (lyophilized powder, material number: L3908, genetic A and B mixture, LGAB), bovine serum albumin (material number: A7030, BSA), poly (methyl methacrylate) (average molecular weight 15,000 Da, material number: 200336, PMMA), phosphate buffered saline (pH 7.4, material number: 806552, PBS) and hydrochloric acid (1.0 N solution, material number: H9892, HCl) were purchased from Sigma–Aldrich (St. Louis, MO). All chemicals were used as received without further purification.

### Preparation of BSA and LGAB solutions and their fibrils

Native proteins were dissolved in distilled water with pH adjusted to 7 and 2. Solvent pH was adjusted to the desired value by adding 1.0 N HCl for pH 2 or PBS buffer for pH 7 and by accurately monitoring using a pH meter (Mettler Toledo, FiveEasy). Protein concentrations were varied from 0.1 (1 mg/ml) to 3 wt.% (30 mg/ml). Protein solutions were stored for 12 h at room temperature prior to any measurements. Fibrils of BSA and LGAB were made by incubating the pH 2 protein solutions in a 90 °C water bath for 12 h followed by rapid quenching of the solution in an ice bath for 1 h. The concentration used for the fibrillization was 3 wt.% (30 mg protein in 1 ml solvent).

### Attenuated total reflection Fourier transform infrared (ATR-FTIR) spectroscopy

Secondary structure of heated and unheated protein solutions were studied using ATR-FTIR. A Fourier-transform spectrometer fitted with a diamond attenuated total reflection accessory crystal (Platinum-ATR, Bruker, Germany) and a mercury cadmium telluride detector was used to collect infrared (IR) spectra at room temperature. Coadded 300 scans covering a wavenumber range of 400–4000 cm^−1^, were collected using a spectral resolution of 4 cm^−1^ by the OPUS™ software. Approximately ~ 10 µl solutions were pipetted on to the crystal and waited for 10 min to achieve a steady state intensity prior to the measurement. During IR spectra processing, corresponding solvent backgrounds were subtracted along with baseline correction and 13-point smoothing using routines provided by the OPUS software. Proper background subtraction was ensured by zero absorbance in the 1720–1850 cm^−1^ region.

Peak deconvolution of amide I band (1600–1700 cm^−1^) of proteins were carried out using OriginPro 2021 software (OriginLab Corporation, Northampton, MA, USA). The Gaussian peak-fit model for each spectrum was based on the Fourier self-deconvolution and second derivative analysis (Additional file 1: Figure S1). To distinguish native and fibrils and to compare β-sheet structures between protein solutions, peak fitting was restricted to sub-peak center wavenumber ranges and two peak ratios [Eqs. (1) and (2)]. Designation of wavenumber ranges was based on Table 2.

**Table 2:** List of vibrational frequency bands in Amide I region of common protein secondary structures in aqueous solution. Information is collected from references [72–74]

Protein secondary structure	Amide I frequency (cm^−1^)
Side chain moieties	1600–1616
Extended chains/β-sheets, aggregated strands	1610–1630
Extended chains/β-sheets/short-segment chains connecting the α-helical segment	1630–1640
Random coil	1637–1648
α-Helix	1648–1660
β-turns	1665–1685
Antiparallel β-Sheets	1675–1695

Prior studies have shown that an antiparallel β-sheet structure is characterized by a low wavenumber, high intensity peak around 1610–1640 cm^−1^ and a high wavenumber, low intensity band between 1675–1695 cm^−1^ [71]. Simultaneous presence of both bands is assigned to oligomers with an antiparallel β-sheet structure, while the presence of only the low wavenumber band (1610–1640 cm^−1^) can be attributed to fibril’s parallel β-sheet structure. Therefore, oligomer index [(Eq. 1), where ${a}_{1675-1695}$ and ${a}_{1610-1640}$ are area under the peaks present anywhere between 1675 to 1695 cm^−1^ and 1610–1640 cm^−1^ respectively], will be the most useful criterion for the characterization of oligomers.

1 $${\text{Oligomer index }}\left( {{\text{OI}}} \right){:}\;\frac{{a_{1675 - 1695} }}{{a_{1610 - 1640} }}$$

The total β-sheet content (Eq. 2) in the protein is estimated by taking the ratio of the area under the curve of the peak centered anywhere between 1610–1640 cm^−1^ (${a}_{1610-1640}$) to the area under the curve for the amide I band (${a}_{1600-1700}$).

2 $${ {\upbeta} \text {-sheet} }\left( \% \right){:}\;\left[ {\frac{{a_{1610 - 1640} }}{{a_{1600 - 1700} }}} \right] \times 100$$

### Small- and wide-angle X-ray scattering (SWAXS)

Low energy, monochromatic X-ray scattering experiments were performed using a SAXSpace system (Anton Paar, Graz, Austria) equipped with a copper (Cu) anode, XRD Eigenmann GmbH PW 2273/20 and Mythen micro-strip X-ray detector (Dectris Ltd., Baden, Switzerland). The X-ray source was operated at 40 kV and 50 mA and water cooled. The line-focus camera used Cu–Kα radiation with a wavelength, *λ* = 0.154 nm (${\text{E}} = \frac{hc}{l} = 8\;{\text{keV}}$) and the irradiation beam was line collimated. A semitransparent beam stop attenuated the highly intense primary X-ray beam, and the primary beam is used to determine the zero-angle or zero-wavevector (${q}_{0}, q=\frac{4\pi }{\lambda }{\text{sin}}(\theta )$, where $2\theta$ is the scattering angle) position and to compensate fluctuation in primary beam intensities and sample absorption. Sample to detector distance was 317 mm and 121 mm for SAXS and WAXS configurations respectively. The scattering vector was calibrated with silver behenate and for current measurements the minimum accessible scattering vector (${q}_{min}$) with SAXS mode was 0.12 nm^−1^.

For SAXS studies, protein solutions and dispersions were filled in the vacuum-tight, 1 mm quartz capillaries, which were thoroughly cleaned using an alkaline solution (Hellmanex® III, powerful alkaline concentrate) and distilled water. Scattering from corresponding solvent filled capillaries were used as the background during background subtraction. WAXS measurements were conducted on solid proteins packed in a plastic adhesive tape. Solid samples were prepared by drying protein solutions at room temperature. Scattering from adhesive tape was subtracted during background correction. Three separate recordings each with an exposure time of 600 s and 300 s were averaged to obtain final scattering profile for SAXS and WAXS respectively. The total exposure time for SAXS on Aβ-42 fibrils dispersed in pH 7 was 1.5 h due to very low protein concentration. The X-ray exposure time for SAXS on α-synuclein dispersion was 2 h. Three measurements were combined using SAXS analysis software (Anton Paar, Austria, version- 4.20.048).

Scattering patterns were corrected with respect to the primary beam position. The relative intensity of scattering data was further corrected using the transmittance of the direct X-ray beam followed by background subtraction. WAXS data were linearly binned to 100 bins. Slit smeared WAXS data were de-smeared using Lake algorithm [75]. All standard corrections were performed using Anton Paar SAXSanalysis software (Austria, version- 4.20.048).

### Spectral small- and wide-angle X-ray scattering (sSAXS)

sSAXS is comprised of high energy, polychromatic X-ray source (tungsten anode, MXR-160/22, COMET), pinhole collimation and spectroscopic photon counting detector (HEXITEC, Quantum Detectors, Oxfordshire, UK). High energy X-rays are used in sSAXS to study thick objects (> 3 mm) and the systems allows to fully utilize the available X-ray flux in the choice of energy range without filtering the incident beam. Polychromatic X-rays were generated using 80 kVp tube voltage and 1 mA current and X-ray exposure time of 300 s. X-ray beam was collimated using 2 mm diameter pinhole collimator made of lead. Sample holders were directly adhered on the collimator and the sample to detector distance (SDD) was 350 mm. The space between source to detector was non-vacuumed. Cadmium telluride (CdTe) pixelated detector is 1 mm thick. The detector has 80 × 80 pixels with 250 µm pixel pitch, where each pixel can measure the energy spectrum. Scattered photons were collected between 30–80 keV using 27 keV energy threshold, 1 keV energy binning and 300 s X-ray exposure time. The wavevector, $q$ is calculated for each scattering angle (2θ) and energy $E$ using the equation:

3 $$q\left( {E,\theta } \right) = \frac{4\pi E\sin \theta }{{hc}}$$

where, *hc* = 1.24 eV-µm. The charge sharing discrimination (CSD) was applied for charge sharing correction [76]. A beamstop with a 300 µm hole was placed in front of the detector to reduce the spectral degradation from pulse pile-up. Energy dependent transmission factor was calculated by measuring primary beam with and without sample under same experimental conditions. For each measurement, the transmission corrected $q$ data were summed for all energy bins. The final sample scattering profile was obtained after background subtraction. The sample holders were treated as the background. The instrument was calibrated using caffeine powder.

## Results and discussion

We first evaluated the secondary structure and % β sheets of recombinant Aβ-42 fibrils, modified and unmodified BSA and LGAB proteins using ATR-FTIR followed by their nanoscopic structure investigations using monochromatic SAXS and WAXS. The selected model protein aggregates which mimic the X-ray scattering characteristics of disease protein aggregates within the length scales of 0.25- to 2-nm were further used for the performance evaluation of sSAXS.

### Protein secondary conformations of Aβ-42 fibrils and synthetic oligomers and fibrils

Analysis of amide I band (1700–1600 cm^−1^) of FTIR is a sensitive and accurate method to address protein secondary structure due to different hydrogen bonding environments within α-helix, β-sheet, turn, or unordered structures. Table 2 summarizes the amide I frequencies of common protein secondary structures in aqueous solution. Additionally, IR spectra can differentiate aggregated protein forms (oligomer vs. fibril) and parallel and antiparallel β-sheet structures within aggregated proteins from native β-sheet protein structures [47, 71, 77]. In our study, fibril with a parallel β-sheet structure and oligomer with an anti-parallel β-sheet structure were distinguished based on the oligomer index (Eq. 1). Prior studies have shown that the oligomer index is above 0.2, typically between 0.2 and 0.3 for proteins with antiparallel β-sheets including Aβ oligomers, but falls below 0.06 for a parallel β-sheet proteins, including Aβ and α-synuclein fibrils [78, 79].

**Fig. 1:**
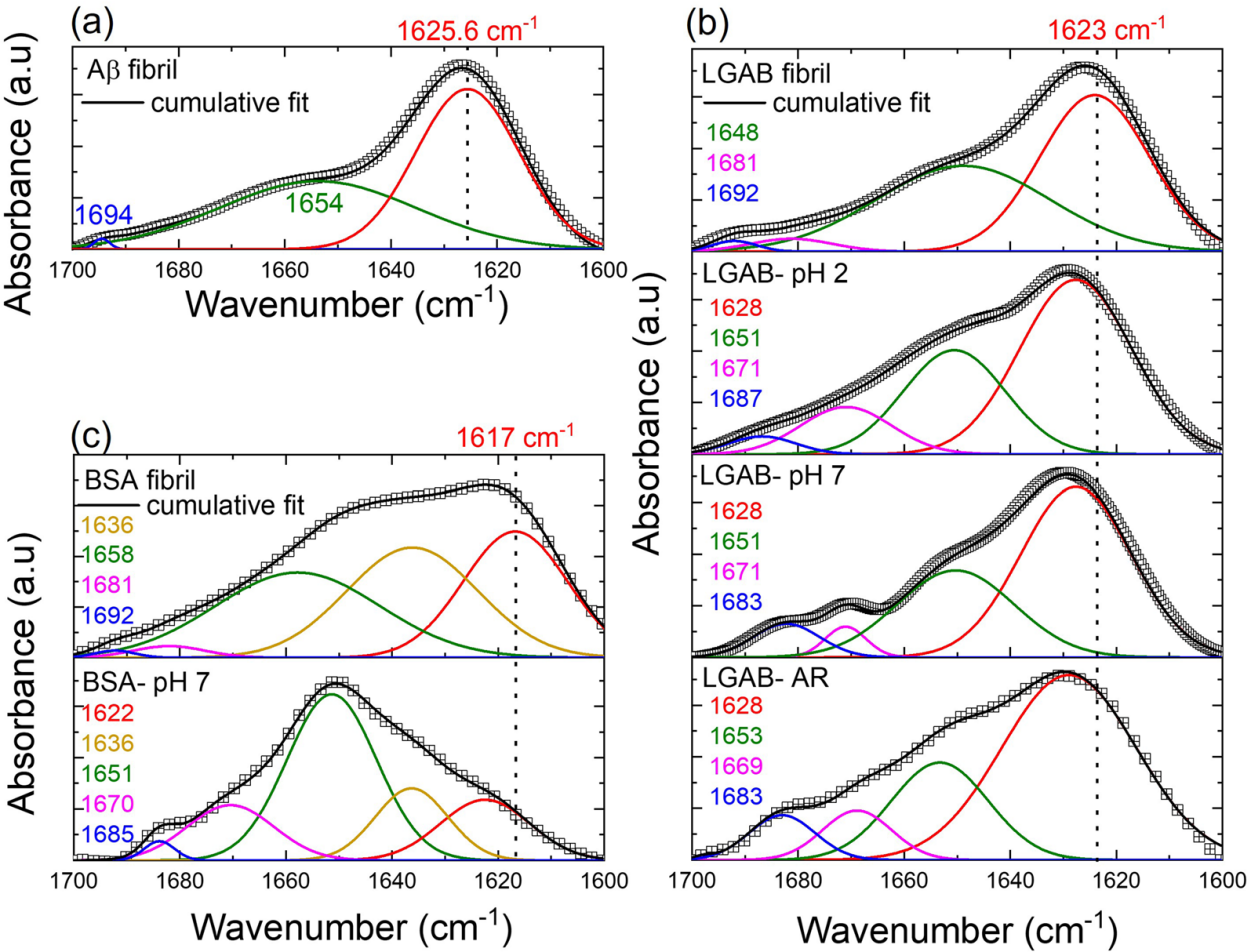
ATR-FTIR spectra in the amide I region (1700–1600 cm^−1^) of (**a**) amyloid fibrils (Aβ-42) formed in vitro (**b**) LGAB as received in the powder form (LGAB-AR), LGAB-AR dissolved in pH 2 and 7 (LGAB-pH2 and LGAB-pH7 respectively) and LGAB treated at pH 2 at 90 °C for 12 h (LGAB fibril) and (**c)** BSA heated at 90 °C for 12 h in pH 2 solution (BSA-fibril) and as received (BSA-AR). Raw data are plotted using symbols. Each spectrum was decomposed by fitting with Gaussian curves. The peak centers (in cm^−1^) of each decomposed peak are indicated in each figure, using the color corresponding to the respective peak color. The identification of protein secondary structure for each wavenumber can be determined based on Table 2. The sum of the fitted curves is displayed as a black continuous line for each. The dotted lines are shown at low wavenumber to facilitate the identification of the major difference between spectra in the β-sheet region. Change in oligomer index with protein modifications can be observed from the change in the area under the curves of blue curves at high wavenumber

ATR-FTIR study on recombinant Aβ-42 fibrils shows the most abundant secondary structure of Aβ-42, which is the parallel β-sheet (55%) with a very small oligomer index (0.01) confirming its fibrillar morphology (Fig. 1a). Prior reports have shown that the fresh wild type peptides exhibit ~ 48% β-sheet content, and that the fibrils resulting from natural mutations lead to an increase in β-sheet content to ~ 58%. This increase correlates with greater amyloidogenicity [80]. The β-sheet structure is promoted by the destabilization of α-helices (1660–1648 cm^−1^) for various systems like Aβ, prion protein (PrP), and the insulin protein [81–83].

To mimic the fibrillar morphology and β-sheet content of A β-42 on commercially available globular proteins, we solution-processed two different and unrelated proteins, LGAB and BSA, at different pH and thermal conditions. The selection criteria for proteins to prepare synthetic aggregates involved identifying those capable of modifying their conformation and nanoscopic structures through physical modifications such as thermal treatments and by tuning electrostatic interactions. Partial unfolding of the protein is considered to precede aggregation and a distinct feature of protein aggregation is the increase of β-sheet structures. The extent of net charge on the polypeptide chain can govern the intermolecular interactions and the final aggregated structure. Therefore, the unfolding of these globular proteins is a complex process that is pH dependent due to its effects on zeta potential starting in a noncooperative transition above 30 °C. The isoelectric point (IEP) of BSA and LGAB lies between pH 4.7 to pH 5.1. The zeta potential increased from ~ − 30 to ~ 30 mV with a decrease in pH from 7.0 to 2.0 (Additional file 1: Figure S2). Previous studies have reported an expanded conformation of BSA at pH 2, and a monomer to dimer transition for LGAB below pH 2.5 that is accompanied by increases in volume and adiabatic compressibility [84, 85]. Therefore, our study limits to two pH values: 2 and 7 (neutral).

β-lactoglobulin is a predominantly β-sheet protein with more than 50% β-sheet content even in the as received powder state without any modifications (Fig. 1b). At pH 2 and room temperature, the increase in LGAB concentration from 0.1 to 3 wt.% shows a change in the shape of amide I band (Additional file 1: Figure S3). vandenAkker et al. have shown that the low concentration peptides (3–4 wt.%) are optimum for the higher persistence length, long and straight fibrils in contrast to higher concentrations (4–7.5 wt.%) [86]. The negative correlation between monomer concentration in one end and β-sheet content, fibril length and persistence length on the other end can lead to more nuclei and shorter worm-like aggregates at high concentrations. Hence, in this study, the protein concentration for fibrillization at pH 2 and 90 °C for 12 h was limited at 3 wt.%. Deconvolution of amide I spectra of 3 wt.% LGAB indicates the percentage of β-sheet is between 50–60% and does not show any specific trend with pH change or with increase in processing temperature (Table 3). However, at room temperature, the oligomer index decreased when pH reduced to 2 (OI = 0.06) compared to neutral pH (OI = 0.12), even though the low wavenumber β-sheet peak position was at 1628 cm^−1^ in both cases.

**Table 3:** Summary of parameters extracted by analyzing ATR-FTIR and solution SAXS spectra of α-synuclein fibril, Aβ-42 fibril and modified and un-modified protein solutions of LGAB and BSA

Sample	Vibrational frequency peak center of β-sheet/ aggregated structure (cm^−1^)	% β-sheet	Particle shape	${R}_{g}$(nm)	a (nm)	b (nm)
α- synuclein fibril- pH 7.4	1630	51 ± 2	Elliptical cylinder		3.5 ± 0.05	13.3 ± 0.2
Aβ-42 fibril-pH 7	1626	55 ± 2	Elliptical cylinder		3.0 ± 0.08	9.2 ± 0.1
LGAB fibril-pH 2	1624	54 ± 3	Elliptical cylinder		1.1 ± 0.03	9.8 ± 0.09
BSA fibril-pH 2	1617	33 ± 5	Elliptical cylinder		1.4 ± 0.2	10.4 ± 0.4
LGAB-pH 2	1628	54 ± 2	Prolate ellipsoid	2.3 ± 0.05	1.5 ± 0.01	3.2 ± 0.02
LGAB-pH 7	1628	55 ± 4	Prolate ellipsoid	2.2 ± 0.01	1.3 ± 0.05	3.3 ± 0.04
BSA-pH 2	1622	20 ± 3	Prolate ellipsoid	3.0 ± 0.02	1.3 ± 0.02	4.6 ± 0.02
BSA-pH 7	1622	18 ± 2	Prolate ellipsoid	2.9 ± 0.01	1.4 ± 0.01	4.5 ± 0.02

Averaged values ± standard deviations are from three independent measurements on fresh samples

However, heating of LGAB solution at pH 2 to 90 °C for 12 h lead to a shift in the β-sheet peak position to a lower wavenumber (approximately 1624 cm^−1^) along with an OI of 0. 02. The different position of the amide I maxima suggests difference in the structure. It has been previously shown that such red-shifting of this β-sheet band in FTIR spectra is due to the presence of intermolecular rather than intramolecular β-sheet structure [87]. This trend is often observed in the spectra of amyloid fibrils relative to the spectra observed for their constituent natively folded proteins. The decrease in OI due to oligomer loss by the reorganization of β-strands from an antiparallel to a parallel β-sheet supports the prediction of LGAB fibrillization. Interestingly, the features of LGAB fibrillization were absent in LGAB-pH 2 solution heated at 45 °C for 13 days (Additional file 1: Figure S4), confirming the requirements of more cooperative transitions for protein fibrillization.

In contrast to LGAB, the unmodified BSA protein is majorly α-helical in nature (50% α-helix and 18% β-sheet), as supported by previous reports [88, 89]. Heating it to 90 °C in pH 2 buffer led to change in IR spectra shape (Fig. 1c). To summarize, there was a clear decrease in α-helical (from 50 to 34%) and β-turn (from 16 to 2%) contents and an increase in β-sheet (from 18 to 33%) and random coil (from 17 to 30%) with a drastic reduction in OI to 0.06. Increase in average molecular size with decrease in pH below the isoelectric point (pH 4.7) and no significant change in dimensions between neutral and IEP were reported previously [84]. This is due to the unfolding of BSA below the IEP. The increased β-sheet content at pH 2 and 90 °C could be due to BSA aggregation and thus the fibril formation. However, compared to BSA fibrils, % β-sheet and OI of LGAB fibrils better match with the recombinant Aβ-42 fibrils.

### Solution small angle X-ray scattering of Aβ-42 fibrils and synthetic oligomers and fibrils

To study the overall structure of proteins and their structural perturbations upon environmental modifications by change in pH and temperature, SAXS (3.2–44.4 nm) and WAXS (0.34–2.1 nm) methods were used. Global alterations in proteins are reflected in the radius of gyration (${R}_{g}$), pair distance distribution ($p\left(r\right)$) and appearance of Kratky plots. Small-angle scattering (X-ray and neutron) and X-ray fiber diffraction (XRD) studies have been reported for brain derived, recombinant and synthetic amyloid or amyloid-like peptides in the presence of solvents [90–92]. We used SAXS to provide information on the fibril cross-section dimension and shape for recombinant Aβ-42 fibrils dispersed in aqueous phosphate buffer at pH = 7 at room temperature.

The 1D scattering profile of Aβ-42 fibrils exhibits classic ${q}^{-1}$ scaling in the low $q$ region characteristic of scattering from a one-dimensional object. The fibrils are modeled as elliptical cylinders. In general, SAXS measures the product of form ($P(q)$) and structure ($S(q)$) factors ($I(q)\propto P\left(q\right)S(q)$). In our model, protein–protein interactions are neglected due to low protein concentration and strong charge screening of the added buffer solvent. Based on prior reports, the fibrils are very long ($L\gg {q}_{min}^{-1}$) and this length cannot be measured with the accessible experimental ${q}_{min}$-value. Based on the studies by Quillin and Matthews [93] and Lattanzi et al. [90], the form factor was factorized into a product of cross-section and cylinder over-all length contributions. The elliptical cylinder cross-section form factor (${P}_{c}(q)$) was written as:

4 $$P_{c} \left( q \right) = \frac{2}{\pi }\mathop \smallint \limits_{0}^{\pi /2} \left( {\frac{{2J_{1} \left( {ql} \right)}}{ql}} \right)^{2} d\varphi$$

where

5 $$l = \left( {\left( {a\sin \varphi } \right)^{2} + \left( {b\cos \varphi } \right)^{2} } \right)^{{{\raise0.7ex\hbox{$1$} \!\mathord{\left/ {\vphantom {1 2}}\right.\kern-0pt} \!\lower0.7ex\hbox{$2$}}}}$$

${J}_{1}(ql)$ is the first order Bessel function of $ql$, $a$, and $b$ are minor and major semiaxes and $\varphi$ is the polar angle.

**Fig. 2:**
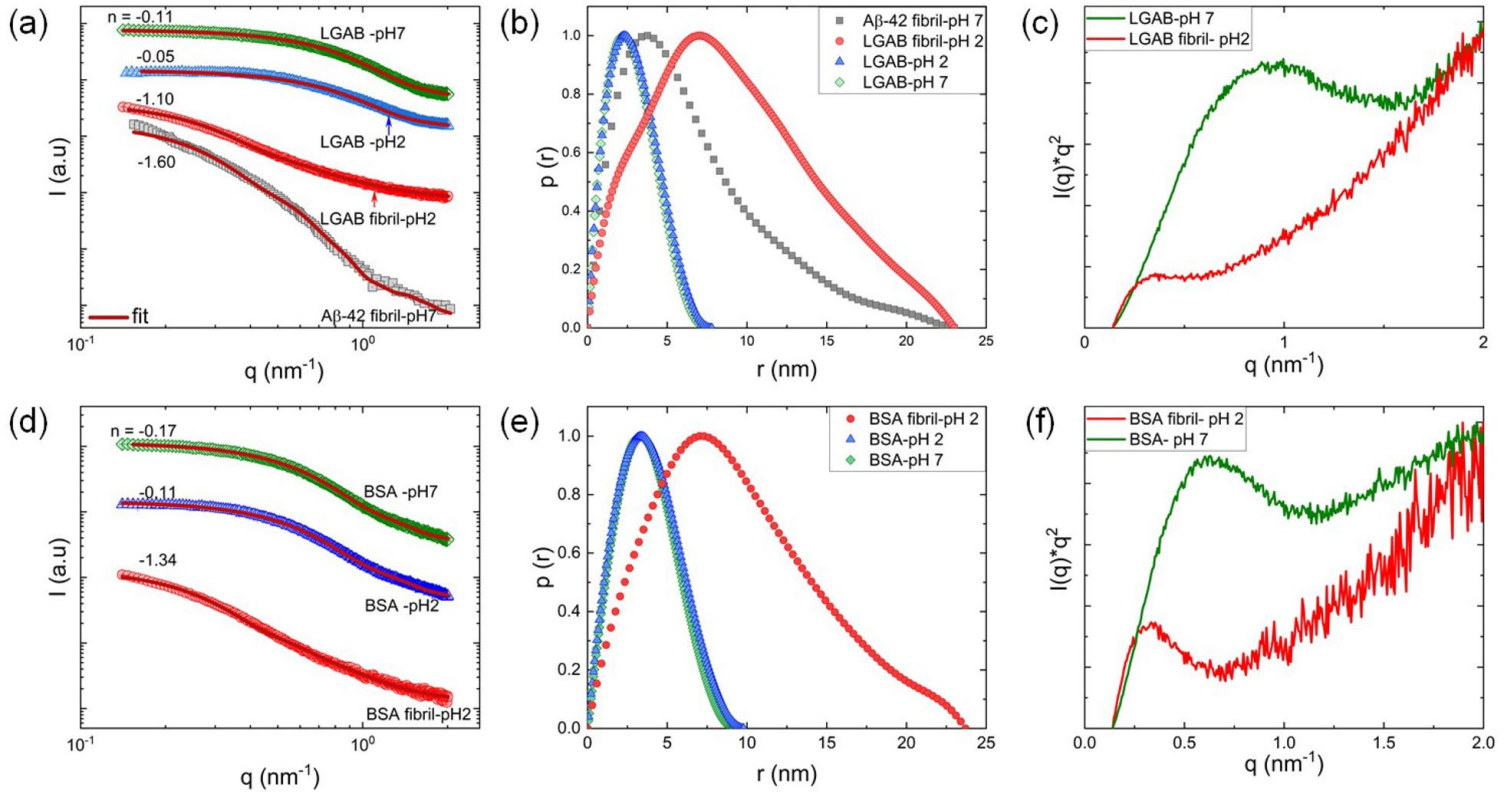
Comparison of structures of Aβ-42 fibrils, modified and unmodified LGAB and BSA using solution SAXS. **a**, **d** 1D scattering profiles where the solid lines are calculated scattering profiles where fibrils are modeled as elliptical cylinders and proteins dissolved in pH 2 and 7 are modeled as ellipsoids. The Y-axis of the curves are scaled for better visualization. **b**, **e** Normalized pair distance distribution ($p(r)$) functions calculated from inverse Fourier transforms of 1D scattering profiles without model predictions. **c**, **f** Kratky plots of LGAB and BSA fibrils compared with their proteins dissolved at pH 7 prior to modifications

The solid line (Fig. 2a) is the modeled scattering profile and *a* = 3 nm and *b* = 9.2 nm described the SAXS pattern well. The values match well with the prior reports based on SAXS and atomic force microscopy (AFM) on mature Aβ-42 fibrils (Table 1). The slight deviation at lower $q$-values could be most likely due to the fibril-fibril attractive interactions arising from hydrophobic interactions. Similar observations were reported for Aβ-42 and α-synuclein [90, 94]. Protein conformational changes and aggregation can be readily assessed from the pair distance distribution function ($p(r)$). This represents the distribution of distances between volume elements inside the particle weighted by the excess density distribution. $p(r)$ was calculated from scattering intensity ($I(q)$) using the indirect Fourier transform method:

6 $$I\left( q \right) = 4\pi \mathop \smallint \limits_{0}^{{D_{max} }} p\left( r \right)\frac{{\sin \left( {qr} \right)}}{qr}dr$$

${D}_{max}$ is the maximum diameter of the particle. The $p\left(r\right)$ of Aβ-42 fibril exhibited a skewed distribution, corroborating the notion of a long rod with a clear maximum at $r$ ~ 4.5 nm corresponding to the radius of the cross section. The information on ${D}_{max}$ for rods is limited by the number of Shannon channels (N_s_), where $N_{s} = D_{max} \left( {q_{max} - q_{min} } \right)/\pi$ [95, 96]. In our study for fibrils, we forced $p\left(r\right)$ to converge to zero at experimentally accessible ${q}_{min}$. Therefore, $p\left(r\right)$ and continuum model fitting reveal an elliptical cross-section for Aβ-42 fibrils.

The scattering intensity of unheated solutions of LGAB and BSA in pH 2 and 7 followed the asymptotic slope ~ 0 at low $q$ region (*q* < 0.3 nm^−1^, Fig. 2a, d). The almost symmetrical shape of $p(r)$ of native proteins resembles the $p(r)$ of sphere (Fig. 2b, e). However, on close inspection, the asymmetry at large $r$ values can be seen, indicating very slight elongation of the spherical species. Therefore, the 1D scattering data of untreated proteins were modelled as prolate ellipsoid and fit parameters are reported in Table 3. The axial ratio ($b/a$ ) of BSA ellipsoids are slightly higher than LGAB ellipsoids. The radius of gyration (${R}_{g}$), the most common descriptor to quantify the overall size of proteins in solution, was determined based on the whole q-range of the scattering curve through $p(r)$ as well as using the classical Guinier approximation ($q{R}_{g}<1.3$).

7 $$R_{g}^{2} = {\raise0.7ex\hbox{$1$} \!\mathord{\left/ {\vphantom {1 2}}\right.\kern-0pt} \!\lower0.7ex\hbox{$2$}}\mathop \smallint \limits_{0}^{{D_{max} }} r^{2} dr$$

8 $$I\left( q \right) \approx I\left( 0 \right)e^{{ - q^{2} R_{g}^{2} /3}}$$

The close agreement between real space ${R}_{g}$ (from $p(r)$) and reciprocal space ${R}_{g}$ (from Guinier region) indicates the absence of big aggregates in unheated protein solutions. Kratky representation was used to easily identify the protein structural compaction. At pH 7, both BSA and LGAB didn’t exhibit a perfect bell shape, instead showing a less compact, partially unfolded profile (Fig. 2e, f).

Prolonged heating of LGAB and BSA at 90 °C and pH 2 led to an increase in the scattering intensity slope to ~ − 1 at low $q$ region (Fig. 2a, d). This reveals the presence of a rod-like structure and confirms protein fibrillization under this condition. The skew shaped $p(r)$ distribution also supports this observation (Fig. 2b, e). The 1D scattering profiles were modeled similarly to the Aβ-42 fibril using the elliptical cylinder cross-section form factor (${P}_{c}(q)$). The model fits matched quite well with the experimental data (Fig. 2a,d). The major and minor semiaxes length scales of LGAB and BSA fibrils are similar to matured Aβ-42 fibril (Table 3). Kratky plots show that thermal treatment decreases the protein compaction, and LGAB exhibited complete unfolding (Fig. 2e, f). However, heated BSA dispersion exhibited a prototypical feature of globular and disordered domains in the partially unfolded construct (Fig. 2f). This could arise from the mixture of fibril and oligomeric states, even though the system is dominated by the fibrillar form as confirmed by $I(q)\sim {q}^{-1.34}$ (Fig. 2d). Therefore, the fibrillization kinetics are faster for LGAB compared to BSA under similar environmental conditions. Our ATR-FTIR study and solution SAXS confirm that the unmodified LGAB and BSA are primarily ellipsoids ($I(q)\sim {q}^{0}$). The dominant aggregate form of model proteins modified at 90 °C and pH 2 is fibrils ($I(q)\sim {q}^{\sim -1}$). Therefore, we did not further purify the system to discern subpopulations as this may better represent the complexity involved in diseased human brain tissue.

### Cross-β sheet structure of Aβ-42 fibrils and synthetic oligomers and fibrils

**Fig. 3:**
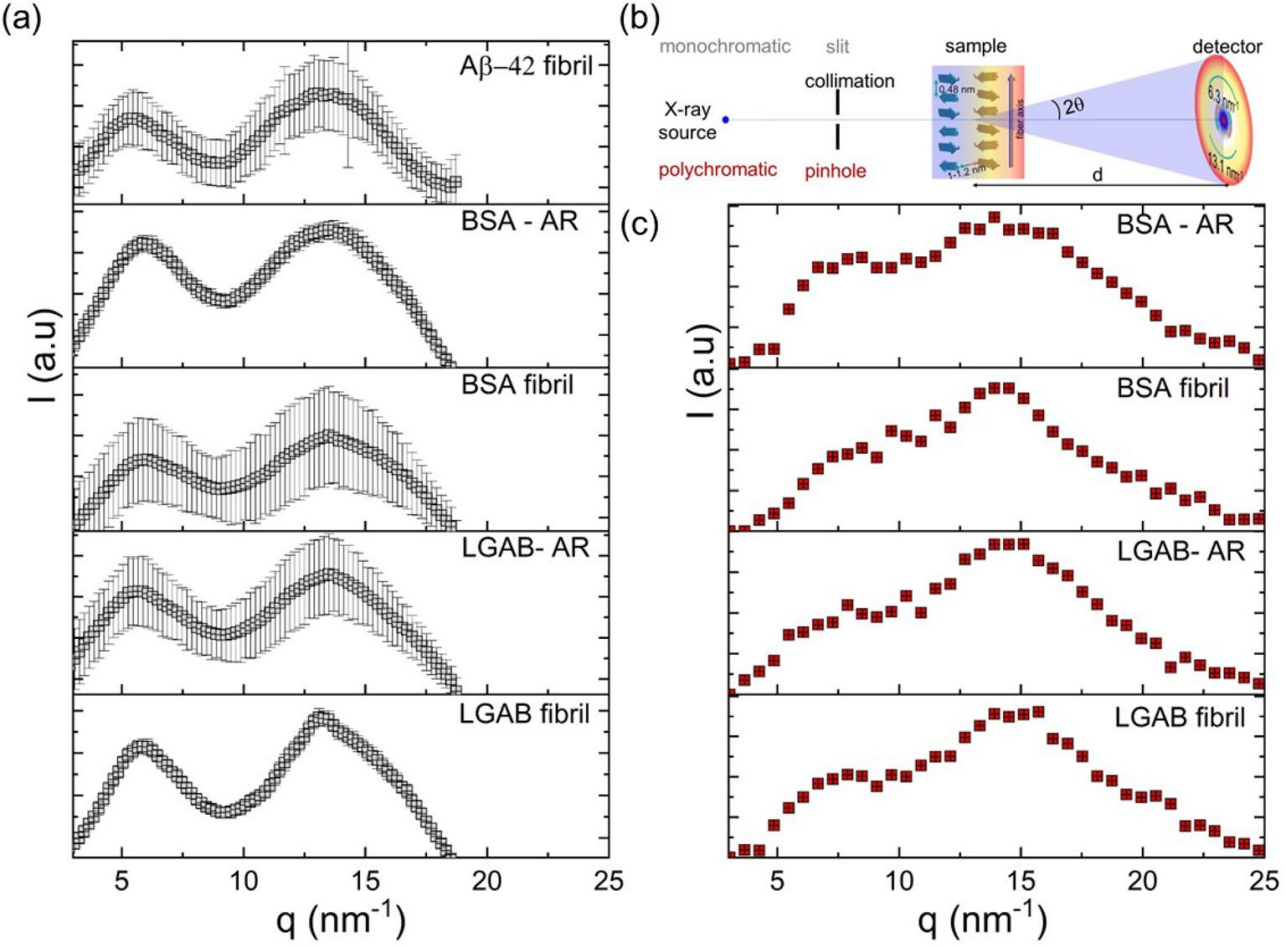
High $q$ (> 3 nm^−1^) scattering profiles of Aβ-42 fibrils, modified and unmodified LGAB and BSA in the powder or flake forms measured from **a** monochromatic, low energy wide angle-X-ray scattering and **c** sSAXS. **b** The differences in the experimental set-ups of monochromatic WAXS and sSAXS are shown in the schematic. sSAXS covers the length scales of 0.25- 2.1 nm. All samples exhibit characteristics of cross-β motifs with reflections ~ $q$= 6.3 and 13.1 nm^−1^

We also collected data on WAXS to clarify whether the modified fibrils have amyloid characteristics as the gold standard for detecting cross-β structures. The principal features of the WAXS profiles of non-oriented fibrils of Aβ-42, LGAB and BSA consist of broad intense peaks at around $q=6.3 {nm}^{-1}$ and $13.1 {nm}^{-1}$, corresponding to Bragg spacings of *d* =  ($2\pi /q$) = 1.0 nm and 0.47 nm respectively (Fig. 3a). These peaks are consistent with the reported spherically averaged cross-β pattern of matured amyloid fibrils (Table 1). The peak at 13.1 nm^−1^ arises from the hydrogen-bonded stacking of β-strands perpendicular to the fiber axis, whereas the 6.3 nm^−1^ peak arises from adjoining β-sheets that run the length of the fibers that are held together by zipper-like side chain interactions between the β-sheets [97]. Interestingly, a qualitatively similar WAXS pattern is observed for unmodified BSA and LGAB spheroids. High $q$ scattering profiles obtained from sSAXS using polychromatic X-ray beam also support these observations. The β-strand separation of 0.47 nm (*q* = 13.1 nm^−1^) is a characteristic feature of parallel and antiparallel β-sheets in folded monomeric proteins as well as in β-sheet aggregates of short peptides. The characteristic distance of 1.0 nm is also observed for these spheroids similar to pattern that arises from the presence of parallel, mature amyloid fibrils (Fig. 3b). FTIR and SAXS studies revealed an antiparallel, prolate ellipsoid structure for unmodified proteins (Figs. 1 and 2). Therefore, the peak at q = 6.3 nm^−1^ is not arising from the presence of mature amyloid-like fibers. More recently, X-ray microdiffraction and solid-state nuclear magnetic resonance studies have demonstrated that soluble, globular, high molecular weight oligomers can adopt either a cross-β conformation containing parallel or an anti-parallel β-sheet arrangement while still being in a globular shape. Stroud et al. have shown that antiparallel Aβ-42 prefibrillar oligomers, with around 30% β-sheet content and the size 13–28 nm in the longest direction, have diffraction pattern similar to those arising from the presence of parallel, mature amyloid fibers [98]. Here, the cross-β structure for fibrils or oligomers are made of adjacent β-sheets that adhere to each other through interpenetration of protein side chains. Gu et.al. have reported similar observations for Aβ-42 oligomers prepared using a fusion protein [99]. We believe the 1.0 nm and 0.47 nm reflections could be arising from the 18 and 55% antiparallel β-sheets present in BSA and LGAB spheroids respectively. Therefore, our study also suggests that the unmodified spheroids can serve as the β-sheet structure mimicking model proteins for amyloid-like oligomers. The detectability of oligomer models using sSAXS is quite promising as an early amyloid detection approach for neurodegenerative diseases. Our study demonstrated the capability of sSAXS to detect up to 18% β-sheet.

### Comparing Aβ-42 fibrils and model aggregates with α-synuclein aggregates

**Fig. 4:**
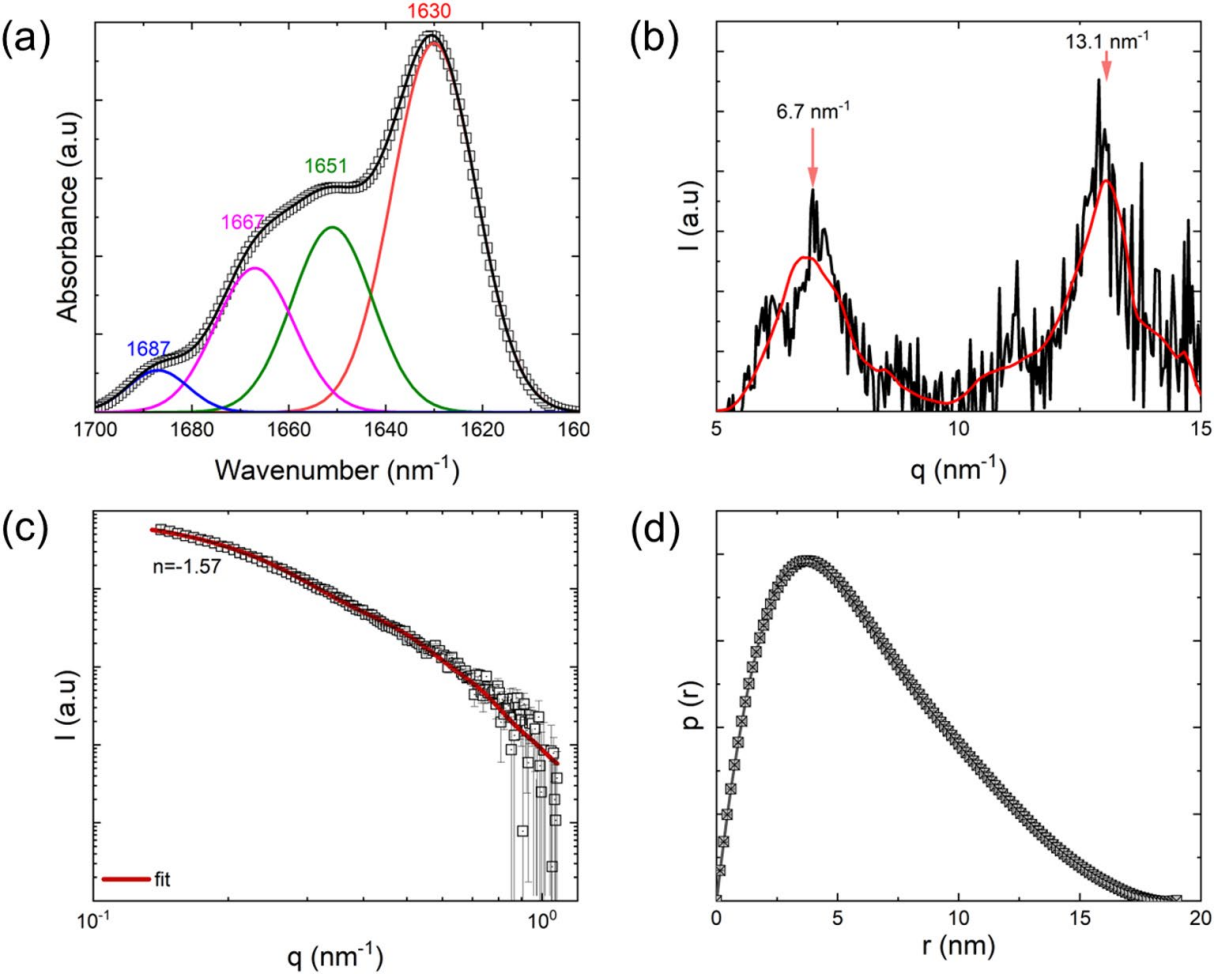
The secondary structure and X-ray scattering properties of recombinant α-synuclein fibrils dispersed in a buffer solution at a concentration of 1 wt.%. **a** The amide I region of the ATR-FTIR spectrum is analyzed, and the secondary structures in this region are deconvoluted using peak analysis. **b** The WAXS profile of 1 wt.% α-synuclein fibrils, dispersed in the buffer solution and exposed to X-rays for a total of 2 h. The denoised spectrum is depicted by the red curve, obtained through wavelet denoising as described in the reference [100]. **c** The 1D SAXS profile of the 1 wt.% α-synuclein fibrillar dispersion. The fibrils are modeled as elliptical cylinders with delta distribution, and the calculated model scattering curve is displayed as the fit. **d** The pair distance distribution function, p(r), of α-synuclein fibrils is illustrated

The wide-angle X-ray scattering of α-synuclein fibrils under neutral pH conditions revealed a distinctive cross-β structure, evidenced by peaks centered at *q* = 6.7 nm^−1^ and 13.1 nm^−1^. These peaks correspond to the spacings between inter β-sheets and inter β-strands, respectively (Fig. 4b). Furthermore, the arrangement of β-strands is observed to be perpendicular to the fibrillar axis. We determined the cross-sectional dimensions of the fibrils by modeling them as elliptical cylinders using an Eq. 4. An elliptical cross-section with a minor axis of a = 3.5 nm and major axis of *b* = 13.3 nm, effectively describes the scattering pattern, as depicted in the fit in Fig. 4c. The radius of gyration (*R*_*g*_) of α-synuclein, purified from human red blood cells, is reported to be 3.3 ± 0.3 nm [101]. This value is nearly double the theoretically estimated *R*_*g*_ for a folded globular protein with 140 amino acid residues, based on the equation $R_{g,theory} = 0.29n^{1/3} ,$ where *n* is the number of amino acid residues, and smaller than that of a random coil with a similar molar mass (~ 5 nm). This suggests that the 3 nm size of α-synuclein could arise from the partially folded intermediate state of the protein chain. The fibril length was not measurable in this experiment because the overall length exceeded the experimentally accessible minimum q range (L > $\frac{1}{{q_{min} }}$). The shape of pair distance distribution function $p(r)$, which is peaked around 3.65 nm, further confirming the long cylindrical morphology of the fibrils (Fig. 4d). The estimation of the maximum pair distance is beyond the detection limit of the current experimental setup; hence, the $p(r)$ is forced to zero at the maximum accessible ${q}_{min}$.

In conclusion, our FTIR, WAXS, and SAXS studies suggest that the secondary structure and nanostructure of α-synuclein fibrils are comparable to Aβ-42 fibrils. Furthermore, the model LGAB fibril mimics the β-sheet content, oligomer index, and X-ray scattering characteristics of both Aβ-42 and α-synuclein fibrils. Additionally, the synthetic fibrils needed to be quantitatively comparable to protein aggregates associated with neurodegenerative diseases in terms of β-sheet content, oligomer index, and cross-sectional dimensions. The structural characteristics of LGAB fibrils, formed by thermally modifying a 3 wt.% (in mg/ml) aqueous solution of native protein adjusted to pH 2 at 90 °C for 12 h, were both qualitatively and quantitatively comparable with recombinant amyloid β-42 fibrils and α-synuclein fibrils.

### Performance evaluation of sSAXS using synthetic oligomers

We additionally evaluated the performance of sSAXS to detect and quantify oligomers in various conditions. These conditions include varying oligomer fractions in tissue-mimicking environment, target oligomer global densities and different packing densities of the deposits. We first evaluated the potential of sSAXS on accurate detection and quantification of different % of β-sheet oligomers from tissue equivalent environment. For this, we selected oligomer-like, unmodified BSA (BSA-AR, β sheet (%): 18) and LGAB (LGAB-AR, β sheet (%): 55). We prepared mixtures by blending protein with different proportions of polymethyl methacrylate (PMMA) powder. Prior studies have shown PMMA as human tissue equivalent material in terms of X-ray attenuation properties [102, 103]. Additional file 1: Table S1 shows similar X-ray mass attenuation coefficients (${\mu }_{m}$, Additional file 1: Eq. S1) of PMMA and brain tissue (white matter and grey matter) in the 30–80 keV energy range. However, we have not compared the X-ray scattering profiles of PMMA and brain tissue.

**Fig. 5:**
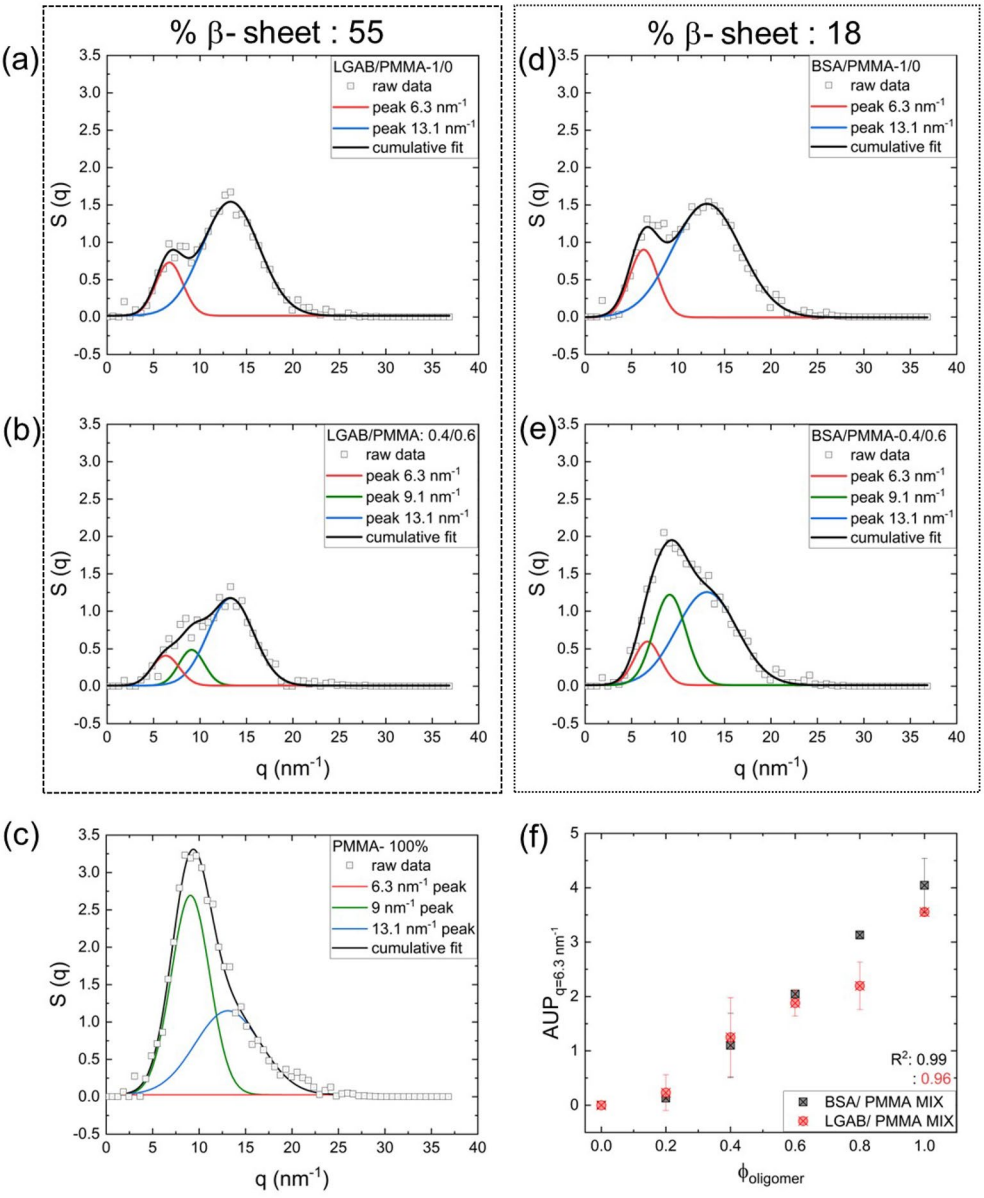
sSAXS calibration using amyloid-like oligomers of LGAB and BSA with % β-sheets of 55 and 18 respectively. sSAXS spectra of unmodified **a** LGAB and **d** BSA oligomer and **b**, **e** their 0.4/0.6 (40% oligomer and 60% PMMA) blends with tissue mimicking polymethyl methacrylate (PMMA). **c** sSAXS spectrum of pure PMMA powder. Spectra of oligomers and PMMA are deconvolved using Gaussian curves. Raw data are shown with symbols and the cumulative fits are shown with black lines. In (**c**) for pure PMMA, a peak center at *q* = 6.3 nm^−1^ is deliberately inserted during deconvolution and the resultant curve indicates the absence of corresponding peak in the raw data. **f** Area under the peak of *q* = 6.3 nm^−1^ (${AUP}_{q=6.3 {nm}^{-1}}$) as a function of oligomer weight fraction (${f}_{oligomer}$) for BSA and LGAB. For each oligomer weight fraction, three independent measurements were performed on freshly prepared samples and the error bars in (**f**) are estimated based on that

In this study, the protein mass fraction (${f}_{protein}$) was varied from 0 to 1. Uniform packing density across blends was ensured by applying a fixed compression stress (Additional file 1: Figure S5). The sSAXS profile of PMMA powder shows a broad peak between *q* *~* 3 nm^−1^ and ~ 25 nm^−1^. Second derivative and Fourier self-deconvolution analyses resolve two overlapped peaks in the scattering spectra. The centers of deconvoluted individual peaks were at *q* *=* 9 nm^−1^ and 13.1 nm^−1^ (Fig. 5c). The incorporation of an artificial peak center at *q* *=* 6.3 nm^−1^ shows the absence of a peak at 6.3 nm^−1^ for PMMA. The deconvoluted sSAXS spectra of PMMA- oligomer blends (PMMA/BSA and PMMA/LGAB) showed three peaks with peak centers at *q* *=* 6.3 nm^−1^, 9 nm^−1^ and 13.1 nm^−1^ (Fig. 5b, e, Additional file 1: Figure S6). The 6.3 nm^−1^ peak solely arises from oligomer β sheet as observed in Fig. 5a and d. The 13.1 nm^−1^ peak is contributed by both β sheet and PMMA. Therefore, the quantification of β sheet in the oligomer/PMMA mix was based on the area under the peak (AUP) of 6.3 nm^−1^ ($AUP_{{q = 6.3\,{\text{nm}}^{ - 1} }}$). $AUP_{{q = 6.3\,{\text{nm}}^{ - 1} }}$ of PMMA blend with BSA or LGAB showed a linear correlation with the protein mass fraction (Fig. 5f). As expected, the $AUP_{{q = 13.1\,{\text{nm}}^{ - 1} }}$ were almost independent of ${f}_{oligomer}$. We believe slight heterogeneity within the PMMA/LGAB blend due to fluffy texture of LGAB powder decreased the ${R}^{2}$ value compared to the BSA/PMMA mix. Most importantly, high ${R}^{2}$ values (> 0.95) validate the potential of sSAXS for accurate β-sheet quantification, even from oligomer-like proteins.

**Fig. 6:**
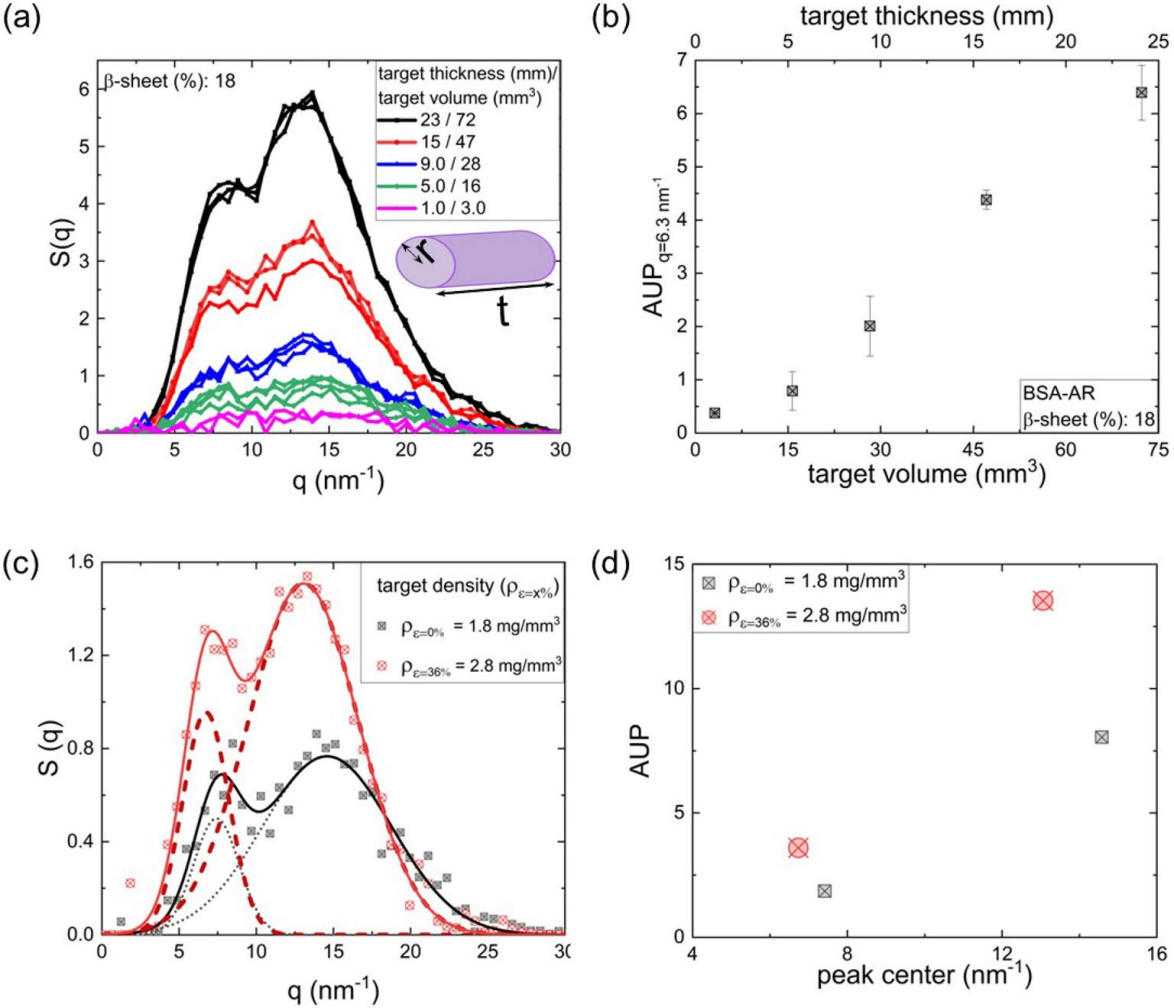
sSAXS performance at different oligomer volumes and packing densities. **a** sSAXS scattering profiles collected at 80 keV, 1 mA, 300 s X-ray exposure for different BSA oligomer (18% β-sheet) volumes. **b** AUP at $q=6.3$ nm^−1^ variation with target thickness and volume **c** sSAXS scattering profiles of two different oligomer packing densities; ${\rho }_{\varepsilon =0\%}=1.8$- and ${\rho }_{\varepsilon =36\%}=2.8$ mg/mm^3^. d) Variation of area under the peaks (AUP) of inter and intra β-sheets at two different packing densities (${\rho }_{\varepsilon =0\%} and {\rho }_{\varepsilon =36\%}$)

We further validated the sSAXS performance at different target volumes by varying the target thickness (Fig. 6a, b). In this study, we used BSA-AR oligomer as the target due to its lowest β-sheet content (18%) among all samples studied here. The polychromatic pencil beam was geometrically focused on a region of interest. Samples were directly placed on a 2 mm pinhole collimator and 2 mm diameter of collimated beam was confirmed using a Gafchromic XR-M2 dosimetry film. Scatter volumes were calculated by assuming a cylindrical target geometry with pencil beam radius and scatter thickness as the radius and height of the cylinder respectively (Fig. 6a inset). Transmission corrected photon count and signal-to -noise-ratio (SNR) decreased with decreasing target volume. $AUP_{{q = 6.3\,{\text{nm}}^{ - 1} }}$ showed almost a linear decrease with scatter thickness ($AUP_{{q = 6.3\,{\text{nm}}^{ - 1} }} \sim t^{0.94}$, Fig. 6b) until $t=5$ mm. Similar trend is observed for AUP calculated for $q=13.1$ nm^−1^ ($AUP_{{q = 13.1\,{\text{nm}}^{ - 1} }}$, Additional file 1: Figure S8). The scattering profile for $t=1$ mm was noisier compared to the profiles of $t\ge 5\mathrm{ mm}$ and the corresponding $AUP_{{q = 6.3\,{\text{nm}}^{ - 1} }}$ shows deviation from the linear scaling.

We further studied the sensitivity of sSAXS to the compaction of oligomer deposits (Fig. 6c, d, Additional file 1: Figure S9 and 10). Loosely bound oligomer deposits showed a shift in peak positions to $q=7.4$ and $14.5$ nm^−1^ with a slightly broader intra β-sheet peak compared to 36% more compact plaque (Fig. 6c and Additional file 1: Figure S10a). The AUP of inter and intra β-sheet peaks showed the dependence on the protein packing density (Fig. 6d). An increase in BSA packing density from 1.8 to 2.8 mg/mm^3^, achieved by applying 36% compressive strain ($\varepsilon \left(\%\right)=-(\frac{{l}_{2}-{l}_{1}}{{l}_{1}})\times 100$,where ${l}_{2}$ and ${l}_{1}$ are oligomer deposition heights in the syringe as shown in Additional file 1: Figure S9), almost doubled the ${AUP}_{q=6.3 {nm}^{-1}}$ ($AUP{\rho }_{\varepsilon =0\%}=1.8$ and $AUP{\rho }_{\varepsilon =36\%}=3.6$). Neuropathology studies of AD brain tissues have described morphologically distinct diffuse and dense core Aβ plaques [104]. Immunohistochemistry, the gold standard for Aβ plaques detection, often fail to identify diffuse plaques [105]. In this context, the sSAXS sensitivity to amyloid packing density and capability to distinct between diffuse and dense deposits are promising. In future, we plan to extend this approach to understand the performance of sSAXS by matching the oligomer and fibrillar plaque densities present in the human brain at different states of the disease.

## Limitations of the study

The current study reports that the oligomer and fibril protein models mimic the secondary structure and nanoscopic X-ray scattering properties of recombinant Aβ-42 fibrils and α-synuclein fibrils. The results were compared with the reported structures of in-vivo protein aggregates of β-Amyloid plaque, α-synuclein, Lewy body, tau tangle, prion and TDP-43 (Table 1). However, the properties of BSA and LGAB model systems beyond these length scales and under other varying conditions in complex in vivo environment are beyond the scope of this work and needs further investigations. The performance evaluation and operating parameter optimizations of sSAXS could also be performed using recombinant oligomer and fibrillar protein aggregates which are commercially available. However, these types of bench testing studies require a large quantity of these expensive recombinant proteins for multiple batch testing and reproducibility studies.

## Conclusions

We propose two globular proteins which can mimic the structural characteristics of β-sheets of fibrillar and oligomeric states of Aβ and α-synuclein within the length scales of 0.35–45 nm. Our FTIR study shows that the combined electrostatic and thermal modifications of LGAB and BSA at pH 2 and 90 °C led to protein unfolding followed by fibrillization with the formation of parallel β-sheet secondary structures. From solution SAXS and powder WAXS, we show that the elliptical cylinder cross-section and cross-β structure of these artificial fibrils matches well the recombinant Aβ-42 fibrils and α-synuclein fibrils. SAXS and FTIR studies conclude that the parallel β-sheet characteristics of LGAB fibrils match more closely Aβ-42 fibrils and α-synuclein fibrils compared to BSA fibrils. Unmodified ellipsoids of LGAB and BSA exhibited antiparallel cross-β structure mimicking Aβ-oligomers. These artificial β-sheets are scalable and inexpensive with easily tunable protein secondary structures and proportions. These model structures may provide important advantages as tractable systems for basic studies evaluating the mechanism of monomer assemblies to various higher order structures during fibrillization and for proof-of-concept studies of structural biomarkers with β-sheets. We used these artificial β-sheet models for evaluating an emerging sSAXS-based method to detect β-sheet rich protein aggregates. Spectral SAXS shows accurate quantification of parallel β-sheets of pure LGAB (average % β-sheet: 54), and BSA (33) fibrils. sSAXS also detected antiparallel cross-β motifs of LGAB (55) and BSA (18) oligomers, a promising technique as this remains challenging for existing neuroimaging modalities to identify oligomers. sSAXS performance evaluation with 18% β-sheet BSA oligomer shows accurate detection of β-sheets from tissue equivalent environment (PMMA) and identification of different target thicknesses. Moreover, sSAXS distinguished different protein packing densities, showing promising for identifying morphologically distinct amyloid depositions. Future work will focus on investigating the detection thresholds of sSAXS under different operational conditions.

### Supplementary Information

**Additional file 1: Figure S1.** IR spectra deconvolution protocol. **Figure S2.** Zeta potential of LGAB and BSA as a function of solution pH. **Figure S3.** FTIR spectra of LGAB dissolved in pH2 at different concentrations varying from 0.1- 3 wt.%. **Figure S4.** FTIR spectra of LGAB-pH 2 solutions heated at 90 °C for 12 h and 45 °C for 13 days. **Table S1.** Values of the X-ray mass attenuation coefficients (${\mu }_{m}$, eq. S1) as a function of photon energy for PMMA and brain tissue. **Figure S5.** Photographs of (a) BSA and (b) LGAB oligomers blended with PMMA powder. **Figure S6.** sSAXS spectra of PMMA/BSA (a-c) and PMMA/LGAB (d-f) blends with oligomer/PMMA proportions (a, d) 0.8/0.2 (b, e) 0.6/0.4 (c, f) 0.2/0.8. **Figure S7.** Area under the peak of $q=13.1 {nm}^{-1}$ (${AUP}_{q=13.1 {nm}^{-1}}$) as a function of oligomer weight fraction (${\phi }_{oligomer}$) for BSA and LGAB. **Figure S8.** Variation of area under the peak (AUP) of $q=13.1 {nm}^{-1}$ with BSA target thickness and volume. **Figure S9.** Photographs of changing the BSA packing density by varying the compressive strain. **Figure S10.** Variation of BSA oligomer peaks full width half maximum (FWHM) and peak height of inter and intra β-sheet peaks of BSA-AR oligomer.

## Data Availability

All data supporting the conclusions of this article are included within the article and in supporting information provided.

## Abbreviations

AD: Alzheimer’s disease
AFM: Atomic force microscopy
AL: Amyloid light-chain
ALS: Amyotrophic lateral sclerosis
ATR-FTIR: Attenuated total reflection Fourier transform infrared
AUP: Area under the peak
Aβ-42: Amyloid β-42
Aβ: Amyloid β
BSA: Bovine serum albumin
BSA-AR: Bovine serum albumin as received
CdTe: Cadmium telluride
CJD: Creutzfeldt-Jakob disease
CSD: Charge sharing discrimination
CSF: Cerebrospinal fluid
FTD: Frontotemporal dementia
FTLD-TDP: Frontotemporal lobar degeneration with TPD-43
IEP: Isoelectric point
LGAB: β-Lactoglobulin genetic A and B mixture
LGAB-AR: β-Lactoglobulin genetic A and B mixture as received
NDDs: Neurodegenerative diseases
OI: Oligomer index
PD: Parkinson’s disease
PET: Positron emission tomography
PMMA: Poly (methyl methacrylate)
PrDs: Prion diseases
PrPs: Prion proteins
SAA: Seed amplification assay
SAXS: Small angle X-ray scattering
SDD: Sample to detector distance
SNR: Signal-to -noise-ratio
sSAXS: Spectral small angle X-ray scattering
SWAXS: Small- and wide-angle X-ray scattering
TDP-43: TAR DNA-binding protein 43
TSE: Transmissible spongiform encephalopathies
TTR: Transthyretin
WAXS: Wide-angle X-ray scattering
XRD: X-ray diffraction
α- Syn: Alpha-synuclein
