# Supplementary material for: Synthetic β-sheets mimicking fibrillar and oligomeric structures for evaluation of spectral X-ray scattering technique for biomarker quantification
Source: Cell Biosci. 2024 Feb 19;14:26. doi: 10.1186/s13578-024-01208-6 (PMC10877803; doi:10.1186/s13578-024-01208-6)
Supplement: Supplementary file 1 — Additional file 1: Figure S1. IR spectra deconvolution protocol. Figure S2. Zeta potential of LGAB and BSA as a function of solution pH. Figure S3. FTIR spectra of LGAB dissolved in pH2 at different concentrations varying from 0.1- 3 wt.%. Figure S4. FTIR spectra of LGAB-pH 2 solutions heated at 90 °C for 12 h and 45 °C for 13 days. Table S1. Values of the X-ray mass attenuation coefficients (${\mu }_{m}$μm, eq. S1) as a function of photon energy for PMMA and brain tissue. Figure S5. Photographs of (a) BSA and (b) LGAB oligomers blended with PMMA powder. Figure S6. sSAXS spectra of PMMA/BSA (a-c) and PMMA/LGAB (d-f) blends with oligomer/PMMA proportions (a, d) 0.8/0.2 (b, e) 0.6/0.4 (c, f) 0.2/0.8. Figure S7. Area under the peak of $q=13.1 {nm}^{-1}$q=13.1nm-1 (${AUP}_{q=13.1 {nm}^{-1}}$AUPq=13.1nm-1) as a function of oligomer weight fraction (${\phi }_{oligomer}$ϕoligomer) for BSA and LGAB. Figure S8. Variation of area under the peak (AUP) of [file 13578_2024_1208_MOESM1_ESM.docx]

Additional Information

**Synthetic β-sheets mimicking fibrillar and oligomeric structures for evaluation of spectral X-ray scattering technique for biomarker quantification**

Karthika Suresh^1,^*, Eshan Dahal^1^, Aldo Badano^1^

^1^Division of Imaging, Diagnostics, and Software Reliability, Office of Science and Engineering Laboratories, Center for Devices and Radiological Health, Food and Drug Administration, Silver Spring, MD 20993, United States of America

*Correspondence to [karthika.suresh@fda.hhs.gov](mailto:karthika.suresh@fda.hhs.gov)


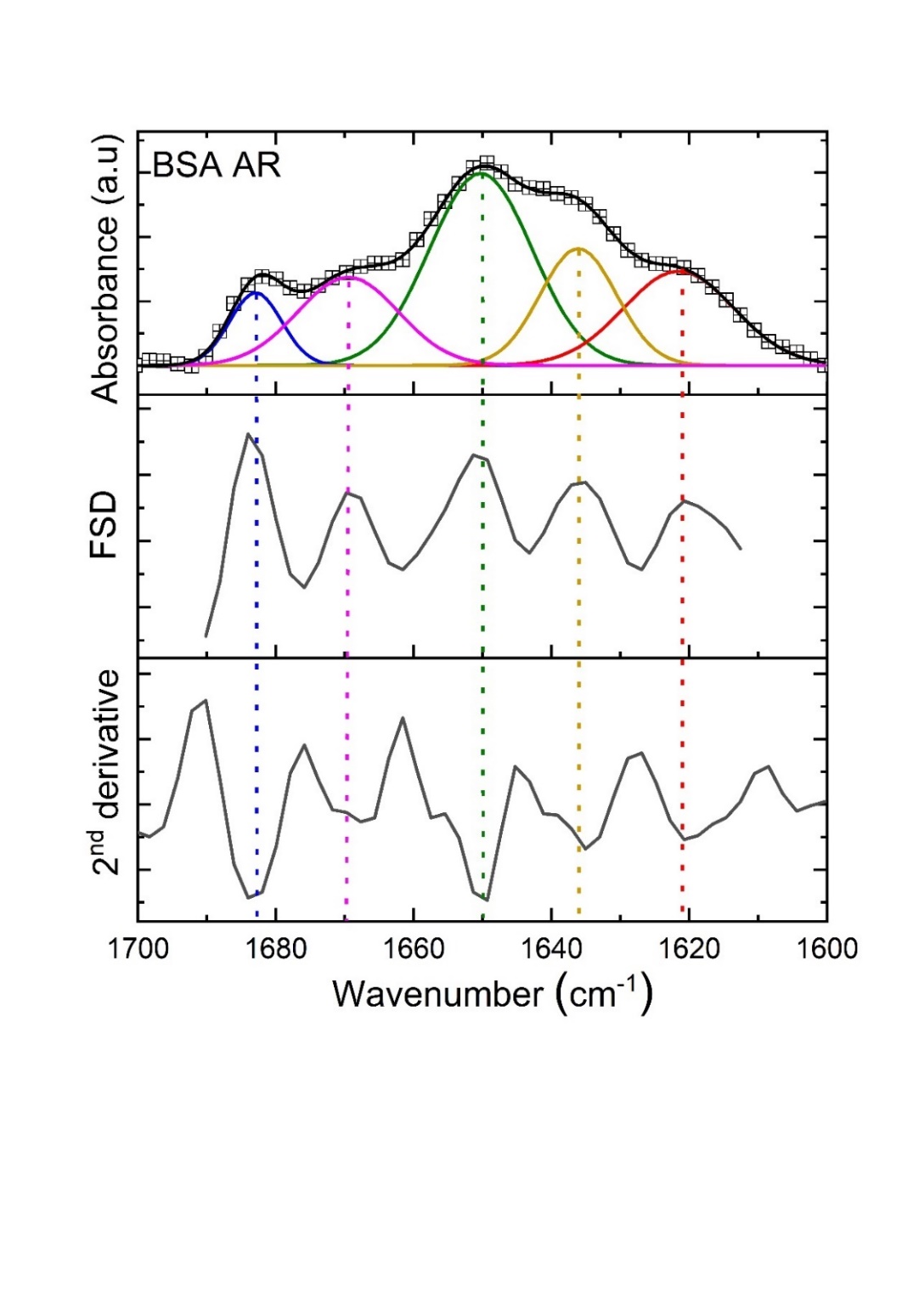


**Figure. S1:** IR spectra deconvolution protocol. The amide I band of BSA-AR is deconvoluted into Gaussian curves at peak positions initially determined by the second derivative and Fourier self-deconvolution (FSD) spectra of the original data.

**Figure. S2:** Zeta potential of LGAB and BSA as a function of solution pH. Data was replotted from references [1,2] .

**Figure S3:** FTIR spectra of LGAB dissolved in pH2 at different concentrations varying from 0.1- 3 wt.%. Shape of spectrum changes between 2-3 wt.%.

**Figure S4**: FTIR spectra of LGAB-pH 2 solutions heated at 90 °C for 12 h and 45 °C for 13 days.

**Table S1**: Values of the X-ray mass attenuation coefficients ($\mu_{m}$, eq. S1) as a function of photon energy for PMMA and brain tissue. The values are taken from NIST X-ray Database and ICRU report 44 [3].

| Material | $\mu_{m}$ (8 keV),  $\left( {{cm}^{2}}/g \right)$ | $\mu_{m}$ (30 keV)  $\left( {{cm}^{2}}/g \right)$ | $\mu_{m}$ (60 keV)  $\left( {{cm}^{2}}/g \right)$ | $\mu_{m}$ (80 keV)  $\left( {{cm}^{2}}/g \right)$ |
| --- | --- | --- | --- | --- |
| Brain grey/white matter | 10.47 | 0.38 | 0.21 | 0.18 |
| PMMA | 6.49 | 0.30 | 0.19 | 0.18 |

X-ray mass attenuation coefficient ($\mu_{m}$):

$\mu_{m}=\frac{\mu}{\rho}=\left( \rho t \right)^{-1}\ln\left( \frac{I_{0}}{I} \right)$ (eq. S1)

where, $\rho$ is the material density, $t$ sample thickness, $I_{0}$ and $I$ are incident and transmitted intensity of monoenergetic photon beam as a function of photon energy respectively.


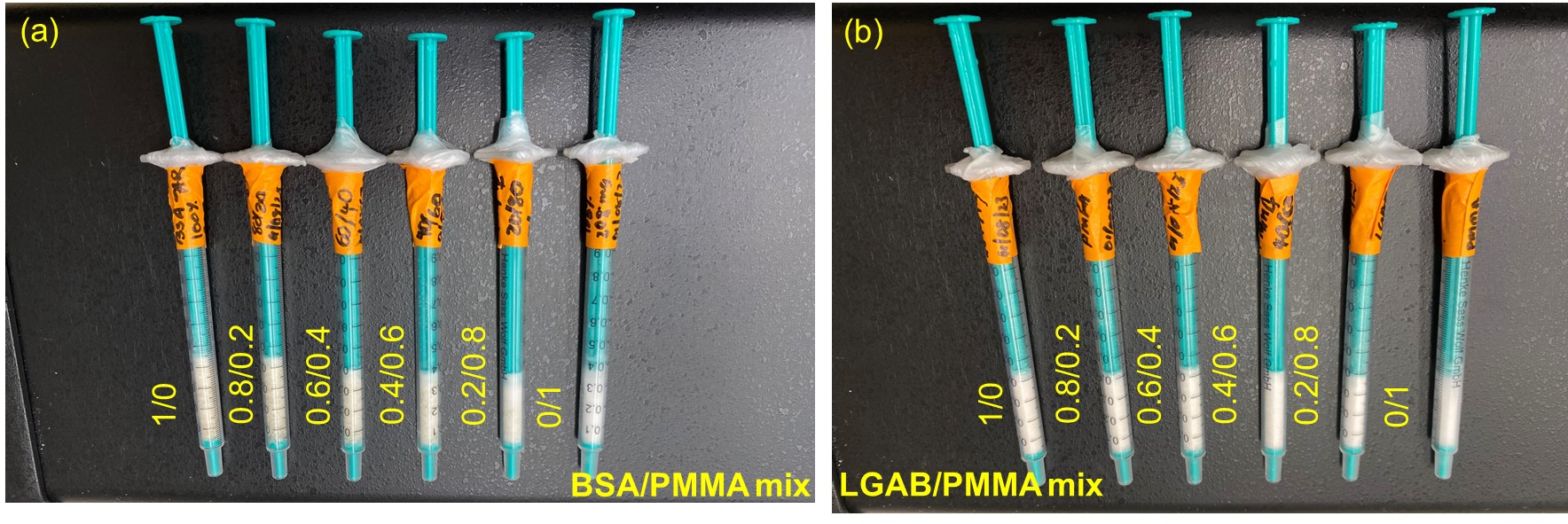


**Figure S5:** Photographs of (a) BSA and (b) LGAB oligomers blended with PMMA powder. Oligomer/PMMA proportions are varied from 1/0 to 0/1. Blends were hand-mixed using a spatula until a homogeneous mixture was formed. Samples for sSAXS measurements were prepared by filling a fixed quantity of blend in a 1 mL plastic syringe. To ensure same packing density across different proportions of blends, blends were compressed to 0.4 mL marking of the syringe and sealed the setup using parafilm as seen in the photograph. Three independent sSAXS measurements were performed for each proportion by exposing the 2 mm size X-ray beam to the 0.1-, 0.2- and 0.3-mL markings of the syringe. LGAB/PMMA mix showed heterogeneity during measurements due to slight settling of PMMA powder.


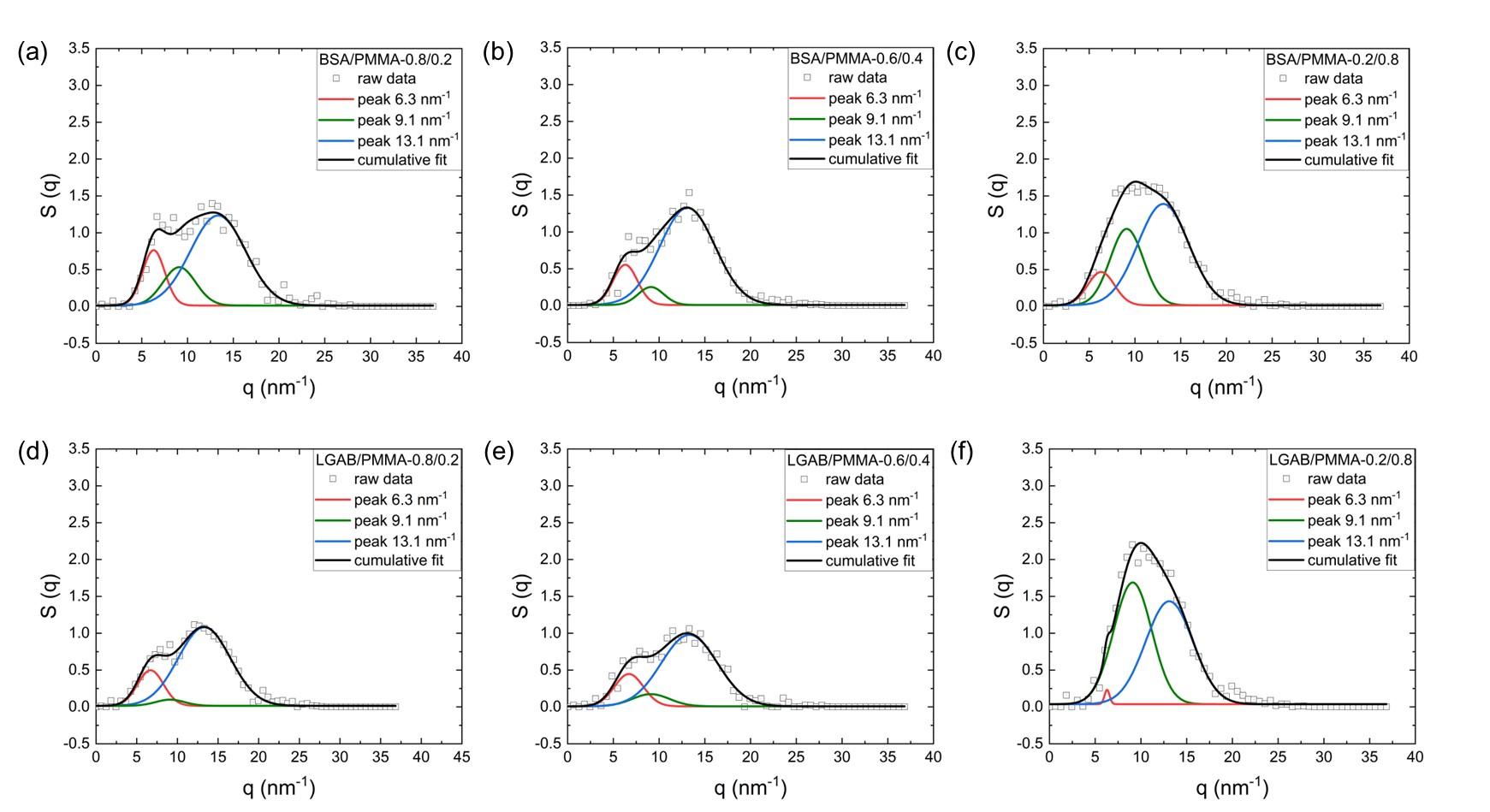


**Figure S6**: sSAXS spectra of PMMA/BSA (a-c) and PMMA/LGAB (d-f) blends with oligomer/PMMA proportions (a, d) 0.8/0.2 (b, e) 0.6/0.4 (c, f) 0.2/0.8. Raw data are shown with symbols and the cumulative fits are shown with black lines. Peaks are deconvoluted using Gaussian curves.


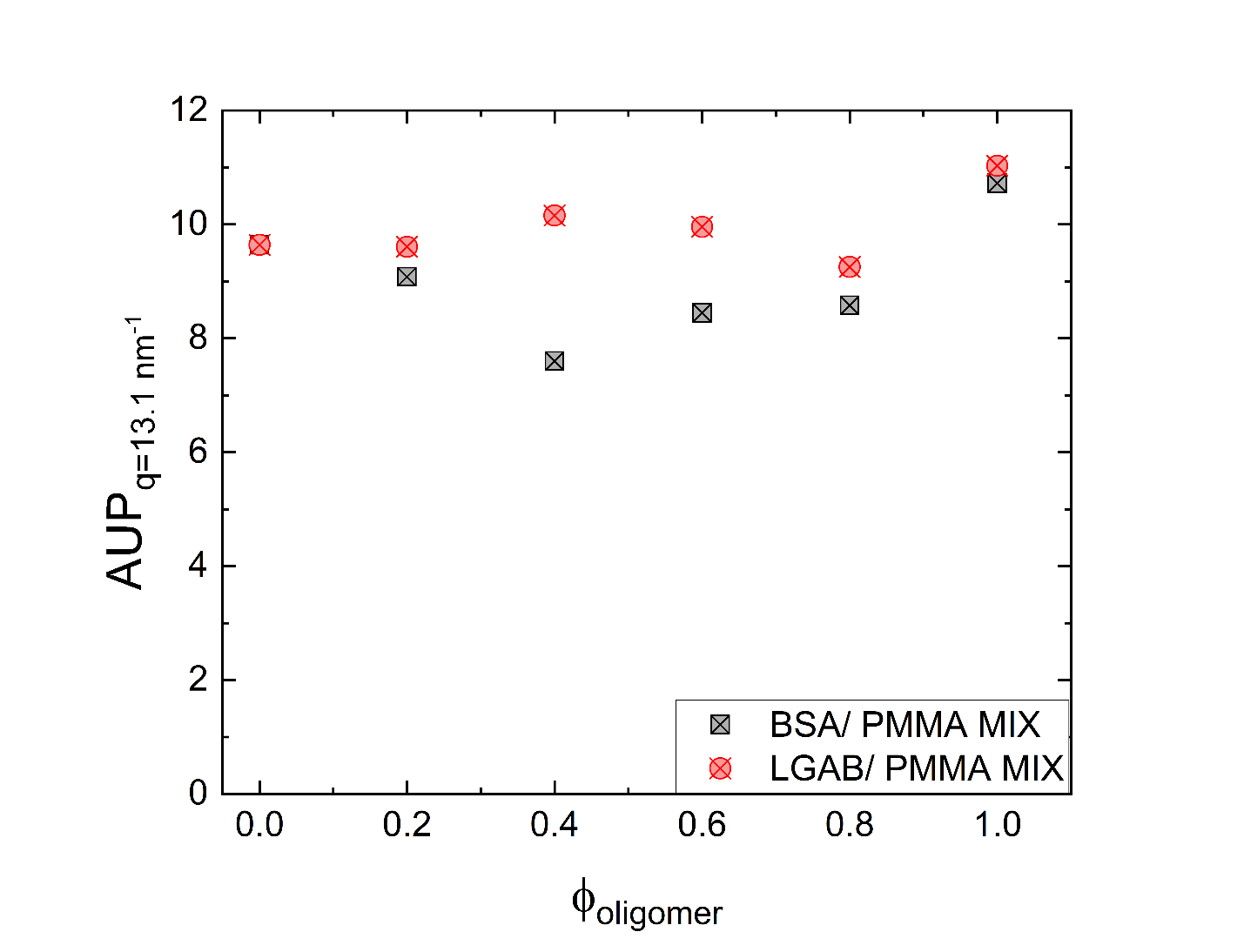


**Figure S7**: Area under the peak of $q=13.1 {nm}^{-1}$ (${AUP}_{q=13.1 {nm}^{-1}}$) as a function of oligomer weight fraction (${}_{oligomer}$) for BSA and LGAB.


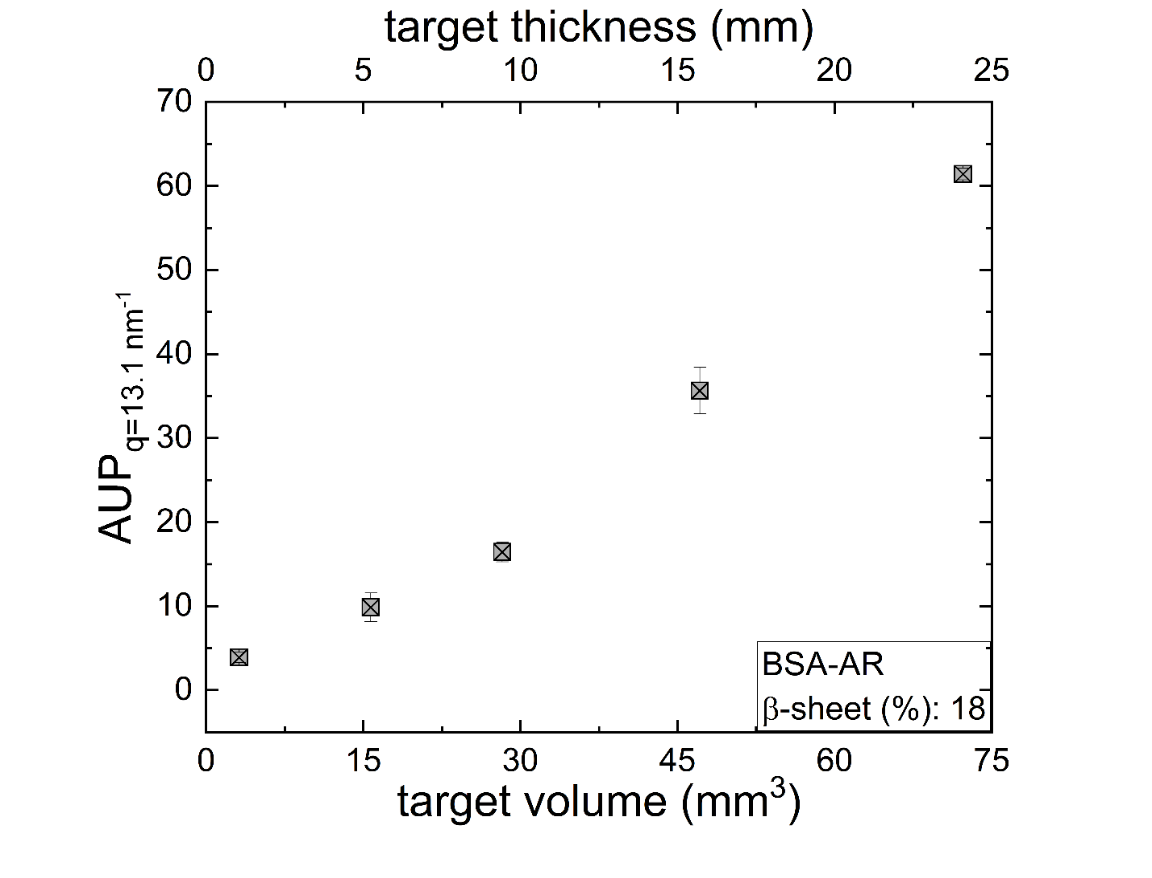


**Figure S8**: Variation of area under the peak (AUP) of $q=13.1 {nm}^{-1}$ with BSA target thickness and volume.


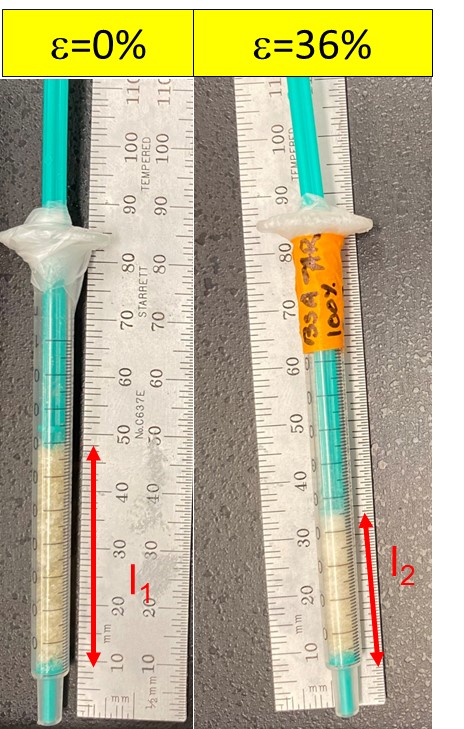


**Figure S9**: Photographs of changing the BSA packing density by varying the compressive strain. 207 mg BSA was filled in 5 mm diameter 1 mL plastic syringe and the height occupied by the sample was 36.3 mm ($l_{1}$). Later height reduced to $l_{1}=23.2 mm$ using the syringe plunger to obtain 36% compression.


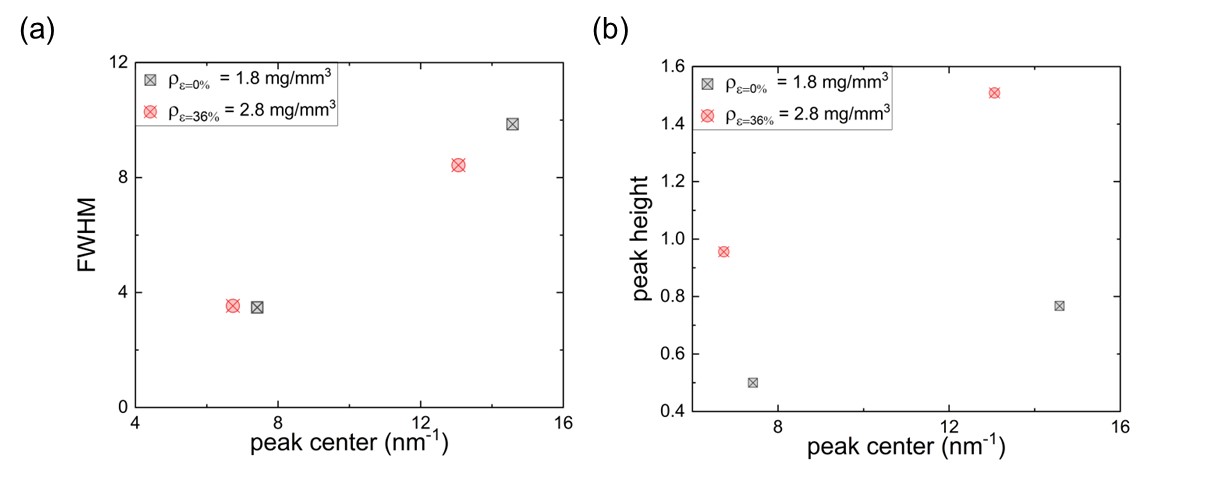


**Figure S10**: Variation of BSA oligomer peaks full width half maximum (FWHM) and peak height of inter and intra β-sheet peaks of BSA-AR oligomer.

**REFERENCES**

1. Engelhardt K, Lexis M, Gochev G, Konnerth C, Miller R, Willenbacher N, et al. pH Effects on the Molecular Structure of β-Lactoglobulin Modified Air–Water Interfaces and Its Impact on Foam Rheology. Langmuir. 2013;29:11646–55.

2. Bukackova M, Rusnok P, Marsalek R. Mathematical Methods in the Calculation of the Zeta Potential of BSA. J Solution Chem. 2018;47:1942–52.

3. Griffiths HJ. Tissue Substitutes in Radiation Dosimetry and Measurement. No. 4. Radiology. 1989;173:202–202.
